# RppH-dependent pyrophosphohydrolysis of mRNAs is regulated by direct interaction with DapF in *Escherichia coli*

**DOI:** 10.1093/nar/gku926

**Published:** 2014-10-13

**Authors:** Chang-Ro Lee, Miri Kim, Young-Ha Park, Yeon-Ran Kim, Yeong-Jae Seok

**Affiliations:** 1 Department of Biological Sciences, Myongji University, Yongin, Gyeonggido 449-728, Republic of Korea; 2 Department of Biological Sciences and Institute of Microbiology, Seoul National University, Seoul 151-742, Korea; 3 Department of Biophysics and Chemical Biology, Seoul National University, Seoul 151-742, Korea

## Abstract

Similar to decapping of eukaryotic mRNAs, the RppH-catalyzed conversion of 5′-terminal triphosphate to monophosphate has recently been identified as the rate-limiting step for the degradation of a subset of mRNAs in *Escherichia coli*. However, the regulation of RppH pyrophosphohydrolase activity is not well understood. Because the overexpression of RppH alone does not affect the decay rate of most target mRNAs, the existence of a mechanism regulating its activity has been suggested. In this study, we identified DapF, a diaminopimelate (DAP) epimerase catalyzing the stereoinversion of L,L-DAP to *meso*-DAP, as a regulator of RppH. DapF showed a high affinity interaction with RppH and increased its RNA pyrophosphohydrolase activity. The simultaneous overexpression of both DapF and RppH increased the decay rates of RppH target RNAs by about a factor of two. Together, our data suggest that the cellular level of DapF is a critical factor regulating the RppH-catalyzed pyrophosphate removal and the subsequent degradation of target mRNAs.

## INTRODUCTION

Nudix (nucleoside diphosphate X) hydrolases are widespread among eukaryotes, bacteria, archaea and viruses and hydrolyze a wide range of organic pyrophosphates. Substrates of nudix hydrolases include nucleoside di- and tri-phosphates, nucleotide sugars and alcohols, dinucleoside polyphosphates (Np_n_N), dinucleotide coenzymes and capped RNAs, with varying degrees of substrate specificity (1). Nudix hydrolases have the conserved 23-amino acid Nudix motif (Nudix box) GX_5_EX_7_REUXEEXGU, where X represents any amino acid and U is usually a bulky hydrophobic amino acid, such as Leu, Val or Ile (2). In *Escherichia coli*, 13 Nudix hydrolase genes have been found, and the physiological functions of some of these proteins were studied genetically and enzymatically (1–6). Among these genes, the *nudH* gene forms an operon with the downstream gene *ptsP* encoding the first component (enzyme I^Ntr^) of the nitrogen-regulatory phosphoenolpyruvate-dependent phosphotransferase system (PTS^Ntr^) (7–9). *nudH* was first identified as a gene associated with the invasiveness of *E. coli* K1 causing neonatal meningitis (10). Genetic experiments with several pathogenic bacteria have shown important roles for NudH and its orthologs in invasiveness and virulence (11–14). Studies on purified NudH have shown that it can catalyze the hydrolysis of diadenosine tetra-, penta- and hexa-phosphates with a preference for diadenosine penta-phosphate (Ap_5_A) (15). However, the physiological importance of this activity is not clear.

Recently, NudH was renamed RppH because it has RNA pyrophosphohydrolase activity (16). RppH cleaves pyrophosphate from the 5′-terminal triphosphate of mRNA and this RppH-catalyzed conversion of the 5′-terminal triphosphate to monophosphate triggers endonucleolytic cleavage by RNase E in *E. coli* and 5′-exonucleolytic degradation by RNase J in *Bacillus subtilis* (16,17). The RNA pyrophosphohydrolase activity is independent of the identity of the 5′-terminal nucleotide *in vitro*, and microarray analysis revealed that *E. coli* RppH induces the degradation of hundreds of transcripts.

The ability of RppH to remove a protective structure at the 5′ terminus is functionally similar to the removal of the cap structure from the 5′ ends of eukaryotic mRNAs. In both cases, the 5′-terminus of the 5′-proximal triphosphate is cleaved to produce a monophosphorylated intermediate vulnerable to attack by a 5′-monophosphate-dependent ribonuclease (16,18). The most well-studied and conserved eukaryotic decapping enzyme is Dcp2 (19). Although Dcp2 shares little sequence homology with RppH, Dcp2 is also a member of the Nudix hydrolase family (20). Whereas many co-factors and decapping enhancers regulating the catalytic activity of Dcp2 have been identified (19), the regulation of RppH activity has not been studied. In this study, we show that DapF, the diaminopimelate (DAP) epimerase catalyzing the biosynthesis of lysine and peptidoglycan (21), forms a tight complex with RppH to stimulate its RNA pyrophosphohydrolase activity both *in vitro* and *in vivo*. Our data suggest that the cellular level of DapF is an important factor regulating the RppH-catalyzed pyrophosphate removal, which is the rate-limiting step for the degradation of several hundred mRNAs in *E. coli*.

## MATERIALS AND METHODS

### Bacterial strains, plasmids and culture conditions

The bacterial strains and plasmids used in this study are listed in Supplementary Table S1. All plasmids were constructed using standard polymerase chain reaction (PCR)-based cloning procedures and verified by sequencing. Bacterial cells were grown as described previously (22). The *rppH* deletion mutant was constructed using *E. coli* DY330 as described previously (23). The *rppH* gene (from the start codon to the stop codon) was replaced by the *neo* gene. The *neo* gene was amplified by PCR from the CR501 strain (22) using the following primers: forward primer, 5′-GCTACCTTTTCGACTATTTCGCGCAGGCGAGTGAGCATAATTGGCGTGACTCAGAAGAACTCGTCACACA-3′ and reverse primer, 5′-GGAGTATGAAACAATCATTCGTATATAAAGCTTTATTTTGAGGTAGTCCGATGATTGAACAAGATGGATT-3′. The PCR product was electroporated into *E. coli* DY330 to generate the strain KM100. The MG1655 Δ*rppH* (KM101) strain was constructed by P1 transduction of the Km^R^ region of KM100.

Strains KM200 (DY330 *dapF*::Tet^R^) and KM201 (MG1655 *dapF*::Tet^R^) were generated similarly. The primers 5′-AGGTGAATTACTAAAAGTCAGTTTCTGTACCCGCGTGATTGGAGTAAATGTTAAGACCCAGTTTCACATT-3′ and 5′-AATATGCTCGCGCTTAACCACTTGCATAAAACCGATATTGGCGCGCTCCGCTAAGCACTTGTCTCCTGTT-3′ were used to amplify the Tet^R^ region from the chromosomal DNA of CR301 (7) and the coding region of *dapF* was subsequently replaced with this *Tet^R^* gene.

To construct pHRppH, an expression vector for His_6_-RppH, primers containing the synthetic restriction enzyme sites for NdeI, located 3 bp upstream of the *rppH* ATG start codon (in boldface type) (5′-GAGGTAGTCAT**ATG**ATTGATGACGATGGCT-3′), and SalI, located 20 bp downstream of the TAA stop codon (5′-ACTATTTCGCGGATCCGAGTGAGCATAATT-3′) (restriction sites underlined) were used to amplify the *rppH* gene from MG1655 genomic DNA. After digestion, the NdeI-SalI fragment was inserted into the corresponding sites of pRE1 (24).

The expression vector pHDapF for the overproduction of His_6_-DapF was generated similarly using a primer pair to amplify the *dapF* gene: forward primer, 5′-GATTGGAGTAACATATGCAGTTCTCGAAAA-3′; reverse primer, 5′-GCAGTTCGGATCCTGGTTGCTTCATAGATG-3′ (engineered restriction sites underlined). The expression vector pHRppH(E56&57A) for the overproduction of His_6_-RppH(E56&57A) was generated similarly using an additional mutagenic primer pair to amplify the region encoding Glu56 and Glu57: forward primer, 5′-GCATAATCCTACTGCAGCAAACAATTCACG-3′; reverse primer, 5′-CGTGAATTGTTTGCTGCAGTAGGATTAAGC-3′ (mutated bases underlined).

### Purification of overexpressed proteins

Purification of His-tagged proteins (His-RppH, His-DapF, His-RppH(E56&57A), His-DapF(C73&217A) and His-EIIA^Ntr^) was performed as previously described with some modifications (7). *E. coli* GI698 strains harboring pRE1-based expression vectors were grown and protein expression was induced as described previously (25). The pellet of cells overexpressing each His-tagged protein was resuspended in binding buffer (50-mM Tris·HCl, pH 8.0, containing 300-mM NaCl) and then passed two times through a French pressure cell at 10 000 p.s.i. The lysate was cleared of cell debris by centrifugation at 100 000 x *g* for 90 min. The soluble fraction was loaded onto a BD TALON^TM^ metal affinity resin (BD Biosciences Clontech) and bound proteins were eluted with binding buffer containing 200 mM imidazole. The fractions containing His-tagged protein were pooled and concentrated in an Amicon Ultracel-3K centrifugal filter (Millipore). To remove imidazole and to purify the protein to homogeneity (>98% pure), the concentrated pool was chromatographed on a HiLoad 16/60 Superdex 75 prepgrade column (GE Healthcare Life Sciences) equilibrated with 50 mM Tris-HCl (pH 8.0) containing 100 mM NaCl. The fractions containing the protein were pooled and concentrated as described above. The purified protein was stored at –80°C until use.

### Ligand-fishing experiments using metal affinity chromatography

*E. coli* MG1655 cells grown overnight in 500 ml of Luria-Bertani (LB) medium were harvested, washed with binding buffer in the presence of 100 μg/ml phenylmethanesulfonyl fluoride (PMSF) and resuspended in 30 ml of the same buffer. The cell pellet was disrupted by passing it twice through a French pressure cell at 10 000 p.s.i. followed by centrifugation at 100 000 x *g* for 60 min at 4°C. The supernatant was divided into aliquots and mixed with either binding buffer as control or 500 μg of His-RppH as bait. Each mixture was incubated with 500 μl of BD TALON^TM^ metal affinity resin in a column for 30 min. The column was washed with 3 ml of binding buffer containing 5 mM imidazole three times, and the bound proteins were eluted with binding buffer containing 200 mM imidazole. Aliquots of the eluted protein sample (10 μl each) were analyzed by sodium dodecyl sulfate-polyacrylamide gel electrophoresis (SDS-PAGE) followed by staining with Coomassie Brilliant Blue R. The protein band specifically bound to His-RppH was excised from the gel, and in-gel digestion and peptide mapping of tryptic digests were performed as previously described (26).

### Surface plasmon resonance spectroscopy

Real-time interaction of RppH with DapF was monitored by Surface Plasmon Resonance (SPR) detection using a BIAcore 3000 (BIAcore AB) instrument as previously described with some modifications (27–29). RppH was immobilized onto the carboxymethylated dextran surface of a CM5 sensor chip. RppH (100 μl, 5 μg/ml) in coupling buffer (10 mM sodium acetate, pH 5.0) was flowed over the sensor chip at 5 μl/min to couple the proteins to the matrix by an *N*-hydroxysuccinimide/*N*-ethyl-*N′*(3-diethylaminopropyl)-carbodiimide reaction (80 μl of mix). Assuming that 1000 resonance units correspond to a surface concentration of 1 ng/mm^2^, RppH was immobilized to a surface concentration of 0.7 ng/mm^2^. The standard running buffer was 10-mM 4-2-hydroxyethyl-1-piperazineethanesulfonic acid (HEPES) (pH 7.2), 150 mM NaCl, 10 mM KCl, 1 mM MgCl_2_ and 0.5 mM ethylenediaminetetraacetic acid, and all reagents were introduced at a flow rate of 10 μl/min. The sensor surface was regenerated between assays by using the standard running buffer at a flow rate of 100 μl/min for 10 min to remove bound analytes.

### Gel filtration chromatography of the RppH–DapF complex

Gel filtration chromatography was performed in an ÄKTA-FPLC system (GE Healthcare Life Sciences). Samples containing 50 μg of RppH, 400 μg of DapF or both proteins in 250 μl of 50 mM Tris-HCl (pH 8.0) containing 100 mM NaCl were incubated for 10 min on ice and injected through a Superose 12 10/300 GL column (GE Healthcare Life Sciences) equilibrated with the same buffer. Gel filtration was performed at room temperature at a flow rate of 0.5 ml/min and the elution profiles were monitored by measuring the absorbance at 280 nm. Fractions of 0.5 ml were collected, and each fraction (20 μl) was analyzed by SDS-PAGE followed by staining with Coomassie Brilliant Blue R.

### *In vitro* assay of RppH activity using diadenosine penta-phosphate (Ap_5_A)

The *in vitro* Ap_5_A-hydrolysis activity of RppH was assayed in a 200-μl reaction mixture containing 50 mM Tris-HCl (pH 8.0), 20 mM MgCl_2_, 100 μM Ap_5_A and 0.5 μg of RppH protein in the presence and absence of DapF (0.5, 1.5 or 7.5 μg). After incubation for 10 min at 37°C, the reaction was terminated by the addition of 20 μl of 20% trifluoroacetic acid. The reaction products generated from Ap_5_A were then identified by high performance liquid chromatography (HPLC) using a Varian dual pump system connected to an ultraviolet-visible detector. A 10-μl reaction mixture was applied to a Hypersil Gold C18 reverse phase chromatography column (Thermo Scientific) equilibrated with 20 mM ammonium acetate buffer (pH 5.0) in water and then chromatographed using a linear gradient of 0–50% 20 mM ammonium acetate in methanol at a flow rate of 1 ml/min for 20 min. The eluted nucleotides were detected at 254 nm.

### *In vitro* assay of RppH activity on synthetic RNAs

Triphosphorylated GCA and ACG mRNAs were synthesized from two strong promoters using the *E. coli* σ^70^-RNA polymerase holoenzyme. DNA sequences near the transcription start sites of the *tyrT* and *rrsA*p1 promoters were slightly modified to make transcription begin with GCAT and ACGT, respectively, as shown in Supplementary Figure S1 and the regions spanning −60 to +25 relative to the transcription start sites were amplified by PCR. The two small mRNAs were synthesized in a 50-μl reaction containing 40 mM Tris-HCl, pH 8.0, 200 mM potassium glutamate, 5 mM dithiothreitol (DTT), 10 mM MgSO_4_, 50 ng of linear DNA template and 10 units of the *E. coli* σ^70^-RNAP holoenzyme. After 1 mM of adenosine triphosphate (ATP), cytosine triphosphate (CTP) and guanosine triphosphate (GTP) were added, the reaction mixtures were incubated at 37°C for 2 h, and the synthesized transcripts were separated by HPLC. Because uridine 5'-triphosphate (UTP) was not added to the reaction, transcription was terminated at the third base. Hydrolysis of small RNAs by RppH was analyzed as described for Ap_5_A except that triphosphorylated mRNAs and their monophosphorylated forms were separated using a linear gradient of 0–20% 20 mM ammonium acetate in methanol for 20 min.

### Measurement of **the phosphorylation state of mRNAs using exonuclease**

The phosphorylation state of mRNAs after the RppH pyrophosphohydrolase reaction was tested using the Terminator 5′-phosphate-dependent exonuclease (Epicentre Biotechnologies), which digests RNAs that have 5′-monophosphate but not triphosphate ends. To test the pyrophosphohydrolase activity of RppH on the *rpsT* P1 transcript, the total RNA (10 μg) isolated from the *E. coli* MG1655 strain was incubated at 37°C for 1 h in reaction buffer containing 50 mM Tris-HCl (pH 8.8) and 5 mM MgCl_2_ with either purified RppH (0.4 or 0.8 μg), DapF (2.5 μg) or both. After phenol extraction, the RNAs were digested with the Terminator 5′-phosphate-dependent exonuclease at 30°C for 3 h followed by northern blotting with an *rpsT*‐specific digoxigenin (DIG)-labeled probe. The decrease in the *rpsT* P1 transcript level corresponds to the level of dephosphorylation by RppH enzyme activity.

### Northern blotting

*E. coli* cells were grown to an OD_600_ of 0.8 in LB medium, and the total RNA was extracted by the hot-phenol method (30). The purity and amount of RNA were verified by electrophoresis on a 1.6% agarose gel followed by staining with ethidium bromide. Northern blot analysis was performed as previously described (31). The total cellular RNA was separated on a 6% urea polyacrylamide gel and transferred to a positively charged nylon membrane (Roche Diagnostics) by electroblotting using the Mini Trans-Blot Cell (Bio-Rad). The blots were hybridized with a DIG-labeled oligonucleotide probe complementary to a specific mRNA at room temperature overnight using the DIG Northern Starter kit (Roche Diagnostics) according to manufacturer's instructions. DIG-labeled probes were prepared by PCR amplification using *E. coli* MG1655 genomic DNA as template, DIG-11-dUTP (Roche) and dNTPs as substrates, gene-specific forward and reverse primers and Ex Taq DNA polymerase (Takara). For the detection of the DIG-labeled probe, the membrane was treated with the anti-DIG-AP antibody and NBT/BCIP (Roche), and the image was scanned using the LAS-4000 IR multi-color imager (Fuji Film).

### RNA half-life measurements

*E. coli* cells were grown to mid-logarithmic phase at 37°C, and the total cellular RNA was extracted at different time intervals after inhibiting transcription by the addition of rifampin (0.2 mg/ml). For quantitative real-time (qRT)-PCR analysis, cDNA was synthesized from 1 μg of DNase I-treated total RNA using cDNA EcoDry Premix (Clontech), according to the manufacturer's instructions. qPCR was performed in triplicate in a reaction volume of 20 μl, which included 10 μl of SYBR Premix Ex TaqII (Takara). Specific products were amplified and detected using the CFX96 Real-Time System (Bio-Rad). The 16S rRNA gene was used as a reference for normalization of the transcript level. The relative expression level was calculated as the difference between the threshold cycle (*C*_t_) of the target gene and the *C*_t_ of the reference gene for each template.

### Bacterial two-hybrid assays

Protein–protein interaction in live *E. coli* cells was assayed using the bacterial two-hybrid (BACTH) system based on the reconstitution of adenylyl cyclase activity as described previously (32). Briefly, the *cya*-deficient *E. coli* strain BTH101 was co-transformed with pUT18C-derived plasmids and pKT25 derivatives. pUT18c and pKT25 encode the T18 and T25 fragments of *Bordetella pertussis* adenylyl cyclase, respectively. The transformants were spotted on LB plates containing 100 μg/ml streptomycin, 100 μg/ml ampicillin and 50 μg/ml kanamycin with 40 μg/ml 5-bromo-4-chloro-3-indolyl-β-D-galactopyranoside (X-gal) as the color indicator for β-galactosidase activity and then incubated at 30°C overnight.

## RESULTS

### Specific interaction between RppH and the DAP epimerase DapF

Although the disruption of the gene encoding RppH affected the stability of hundreds of mRNAs, the overexpression of RppH did not influence the longevity of most target mRNAs (33). This observation suggests the existence of a prior event or another cellular factor regulating the activity of RppH. Genes encoded within the same operon often function in the same physiological context. The *rppH* gene is in the same operon as *ptsP*, which encodes enzyme I^Ntr^ of the PTS^Ntr^, which regulates diverse physiological processes, such as amino acid metabolism and potassium homeostasis, by sensing nitrogen availability (8,9). Therefore, we envisioned that the regulation of RppH might be related to nitrogen metabolism.

**Figure 1.**
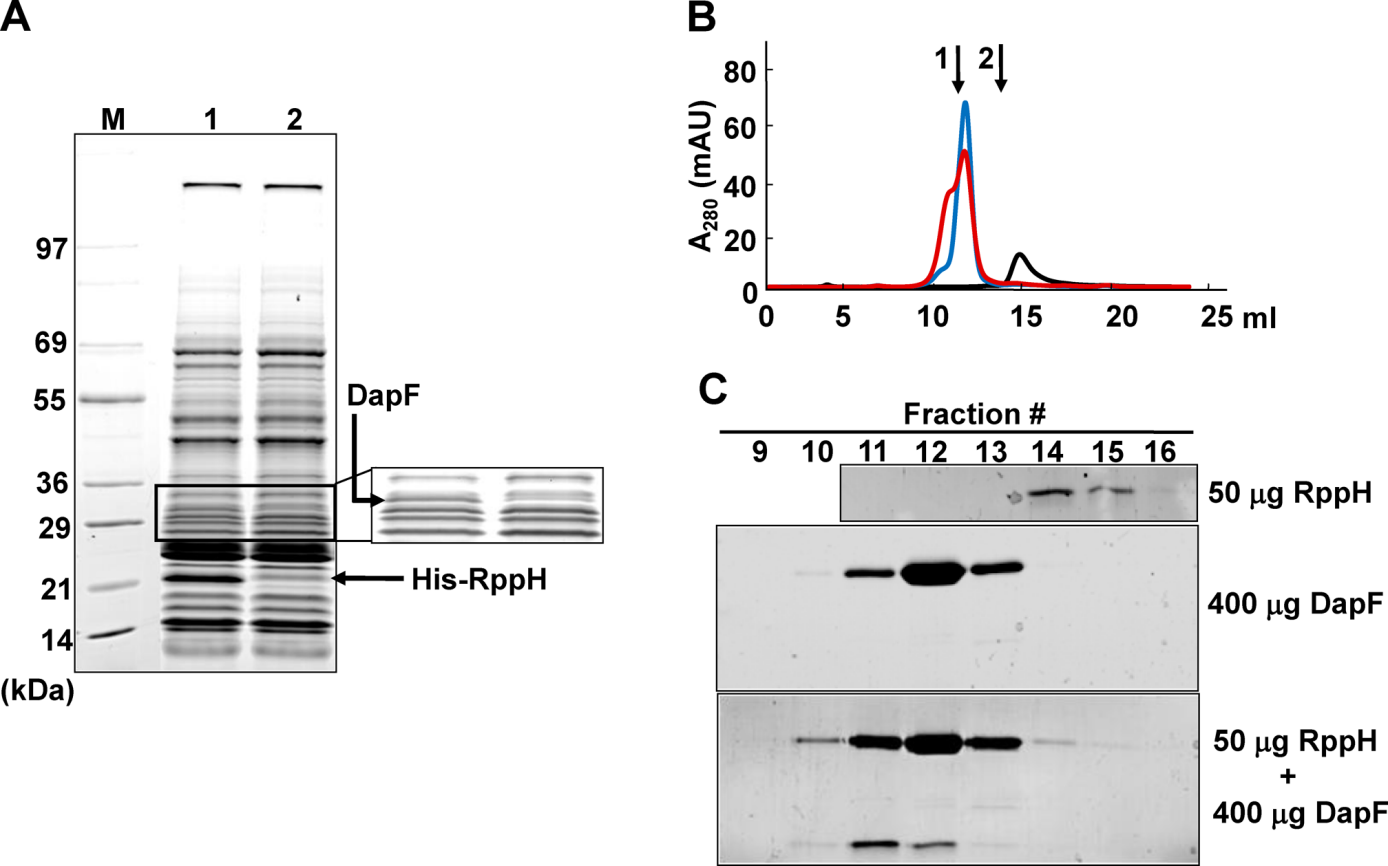
Direct interaction between RppH and DapF. (**A**) Ligand fishing using purified His_6_-RppH as bait. Crude extract prepared from MG1655 grown in 500 ml of LB to stationary phase was mixed with 500 μg of purified His-RppH (lane 1) or binding buffer (50 mM Tris-HCl (pH 8.0) containing 300 mM NaCl) (lane 2). Each mixture was incubated with 500 μl of TALON resin for metal affinity chromatography. The proteins bound to each column were processed as described in the Materials and Methods section. The EzWayTM Protein Blue MW Marker (KOMA Biotech) was used for molecular mass markers (lane M). (**B**) Gel filtration chromatography of RppH, DapF and the RppH–DapF complex. Samples containing 50 μg of RppH, 400 μg of DapF, or both proteins were injected onto a Superose 12 10/300 GL column (GE Healthcare Life Sciences). Gel filtration was performed at a flow rate of 0.5 ml/min and the elution profiles were monitored by measuring the absorbance at 280 nm: black line, RppH; blue line, DapF; red line, the RppH–DapF mixture. (**C**) Fractions (0.5 ml) were collected, and each fraction (20 μl) was analyzed by SDS-PAGE and stained with Coomassie Brilliant Blue R. Arrows 1 and 2 indicate the positions of elution peaks of bovine serum albumin (∼66 kDa) and bovine carbonic anhydrase (∼29 kDa), respectively.

To elucidate the mechanism regulating the activity of RppH, we used a ligand-fishing strategy (34,35). A crude extract from *E. coli* MG1655 was mixed with RppH or a 6His-tagged form of RppH (His-RppH) and subjected to pull-down assays using a metal affinity resin. We observed a protein band (apparent molecular mass of ∼30 kDa) specifically eluting in a fraction containing His-tagged RppH (Figure 1A). Peptide mapping of the protein band indicated that it corresponds to the diaminopimelate (DAP) epimerase DapF, which catalyzes the stereoinversion of L,L-DAP to *meso*-DAP, the penultimate step in the lysine biosynthetic pathway (21,36). In most Gram-negative organisms and some Gram-positive organisms, *meso*-DAP is also used as a component of the pentapeptide in the biosynthesis of peptidoglycan (37).

To confirm the interaction between RppH and DapF, DapF with an N-terminal 6His-tag (His-DapF) was constructed. A crude extract of wild-type MG1655 cells was mixed with purified DapF or His-DapF and subjected to pull-down assays using TALON metal affinity resin. As shown in Supplementary Figure S2A, the protein band corresponding to RppH was detected in the eluate of this wild-type cell extract mixed with His-DapF but not in the eluate of the same extract mixed with untagged DapF. In addition, when ligand-fishing experiments were performed with the crude extract of RppH-overexpressing cells after incubating with DapF or His-DapF, a much higher amount of RppH was pulled down by His-DapF, but not by DapF, as expected (Supplementary Figure S2B). These data support the interaction of RppH with DapF.

To test the specificity of the interaction between RppH and DapF, purified DapF was mixed with varying amounts of His-RppH or the control protein His-enzyme IIA^Ntr^ and subjected to pull-down assays (Supplementary Figure S3). Whereas the amount of the DapF protein bound to the column was independent of the amount of His-enzyme IIA^Ntr^ pre-bound to the affinity resin, DapF binding increased with increasing amounts of His-RppH added to the column, confirming that DapF specifically interacts with RppH. The band intensities of the two proteins (Supplementary Figure S3) suggested that RppH interacts with DapF in 1:1 ratio.

The tight interaction between DapF and RppH was also confirmed by gel filtration analysis. The elution profile of the complex from a Superose 12 gel filtration column (10 × 300 mm) was compared with those of the individual proteins. RppH was eluted with a symmetrical peak at 14 ml, corresponding to the monomeric form (20 kDa), while purified DapF was eluted at ∼12 ml, corresponding to the dimeric form (62 kDa) (Figure 1B). This is consistent with previous studies on *E. coli* and *Haemophilus influenzae* in which the dimeric form of DapF was observed (36,38). When a mixture of the two proteins was loaded onto the column, the elution peak of RppH shifted to ∼11.6 ml (Figure 1B and C), indicating that the molecular mass of the RppH–DapF complex is ∼100 kDa (Supplementary Figure S4), corresponding to a heterotetramer with a 2:2 stoichiometry.

Kinetic parameters for the binding of DapF to RppH were determined using RppH immobilized to a sensor chip in the BIAcore system. Three different concentrations (20, 50 and 70 μg/ml) of purified DapF were used for the binding analysis (Supplementary Figure S5). Using the BIAevaluation 2.1 software, the dissociation constant (*K*_d_) for the DapF–RppH interaction was determined to be ∼5.2 × 10^−9^ M, assuming a dimer–dimer interaction between DapF and RppH.

### DapF stimulates RppH activity *in vitro*

**Figure 2.**
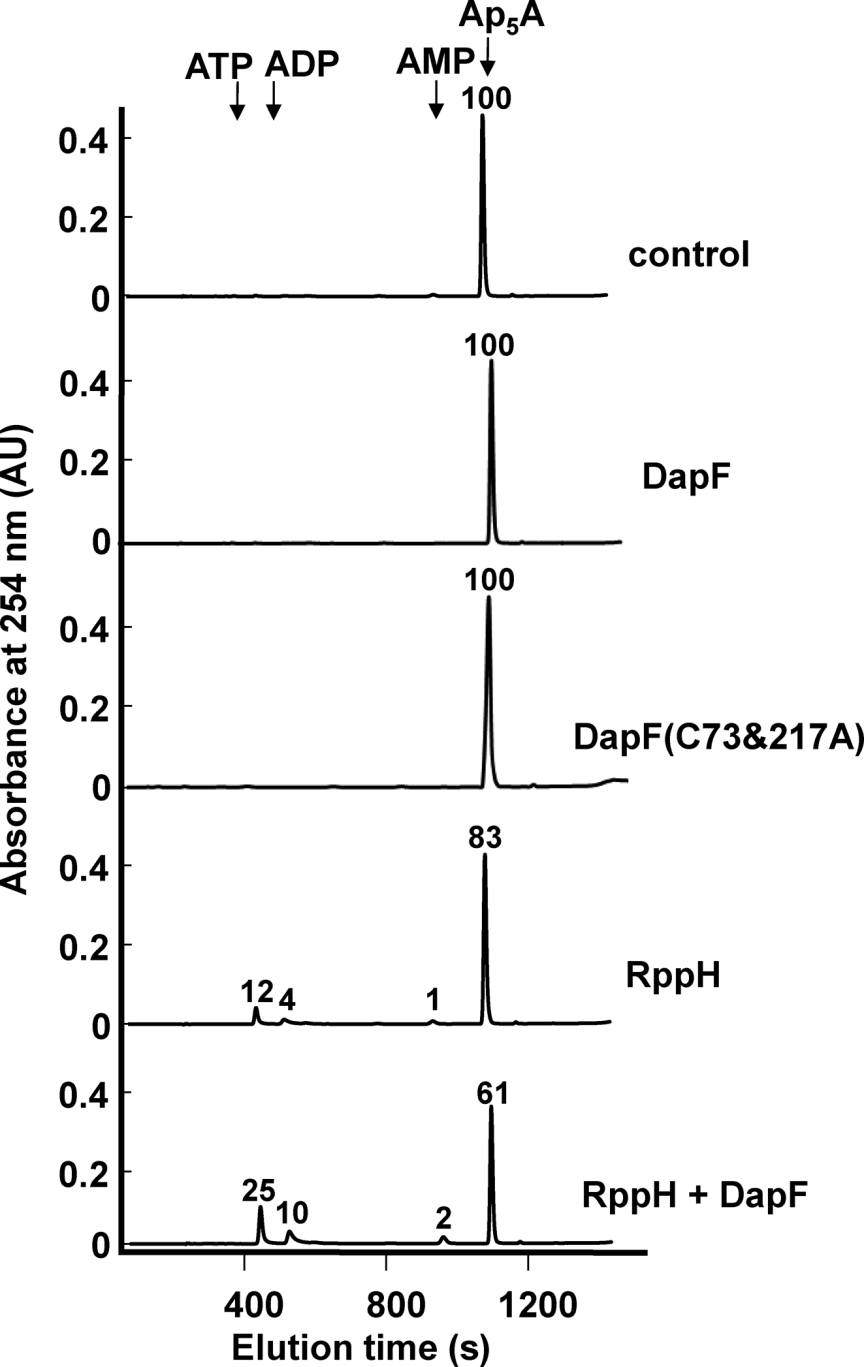
Effect of DapF on the Ap_5_A-hydrolysis activity of RppH. The Ap_5_A-hydrolysis activity of RppH (0.5 μg) was assayed in a 200-μl reaction mixture containing 50 mM Tris-HCl (pH 8.0), 20 mM MgCl_2_ and 100 μM Ap_5_A with and without DapF (1.5 μg). After incubation at 37°C for 10 min, the reaction was terminated by adding 20 μl of 20% trifluoroacetic acid. A 10-μl reaction mixture was applied to a Hypersil Gold C18 reverse phase chromatography column (Thermo Scientific) equilibrated with 20 mM ammonium acetate buffer (pH 5.0) in water and then chromatographed using a linear gradient of 0–50% 20 mM ammonium acetate in methanol at a flow rate of 1 ml/min for 20 min using a Varian dual pump HPLC system. Ap_5_A and the reaction products were monitored by measuring the absorbance at 254 nm (A_254_). Relative peak areas are shown on the top of each peak with the peak of Ap_5_A in the control sample assigned a value of 100.

The tight interaction between DapF and RppH suggested that DapF may regulate the activity of RppH. To test this hypothesis, we examined the effect of DapF on RppH activity. RppH catalyzes the hydrolysis of diadenosine penta-phosphate (Ap_5_A) *in vitro* (15). Consistent with the previous study, we observed that purified RppH hydrolyzed Ap_5_A to ATP, adenosine 5'-diphosphate (ADP) or adenosine 5'-monophosphate (AMP) *in vitro* (Figure 2). Notably, the addition of DapF to the reaction mixture significantly increased the Ap_5_A hydrolase activity of RppH. DapF alone did not show any pyrophosphohydrolase activity, implying that the apparent activation of RppH by DapF does not reflect contamination with RppH during the preparation of DapF.

**Figure 3.**
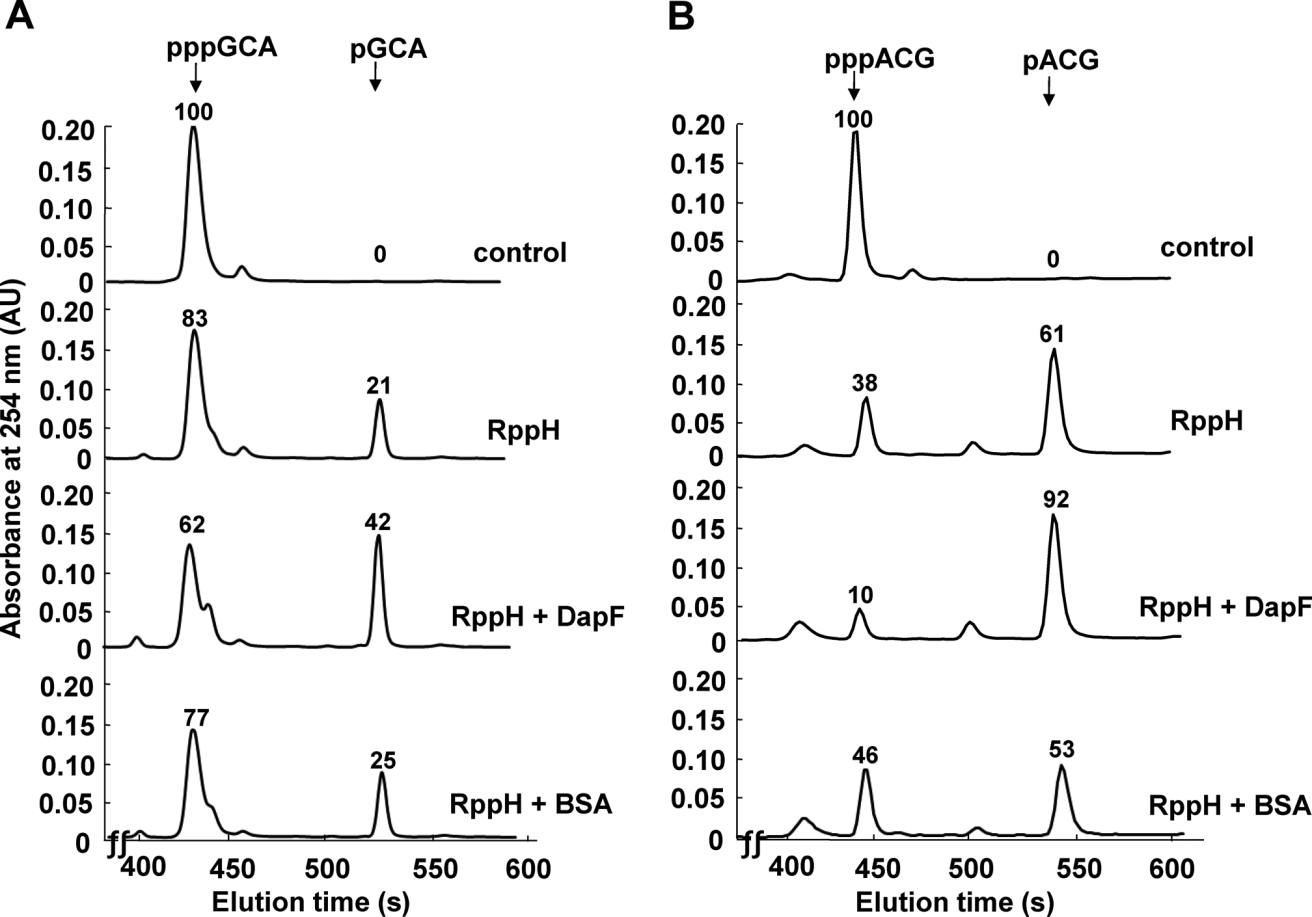
Effect of DapF on RppH pyrophosphohydrolase activity. The pyrophosphohydrolase activity of RppH (0.5 μg) was measured *in vitro* using two synthetic RNAs (pppGpCpA (**A**) and pppApCpG (**B**)) in the presence and absence of 1.5 μg of DapF or BSA (as control). Triphosphorylated and monophosphorylated RNAs were separated on a Hypersil Gold C18 reverse phase chromatography column (Thermo Scientific) equilibrated with 20 mM ammonium acetate buffer (pH 5.0) in water and then chromatographed using a linear gradient of 0–20% 20 mM ammonium acetate in methanol at a flow rate of 1 ml/min for 20 min using a Varian dual pump HPLC system. The eluted nucleotides were monitored by measuring the A_254_. Relative amounts of triphosphorylated or monophosphorylated forms of two synthetic RNAs were measured by the relative peak areas (shown on the top of each peak) with a peak area of 100 for the amount of triphosphorylated RNAs in the control sample.

We then tested the RNA pyrophosphohydrolase activity of RppH using a triphosphorylated short mRNA (5′-pppGpCpA) as a substrate. A recent study showed that *B. subtilis* RppH requires at least two unpaired nucleotides at the 5′ end of its RNA substrates (39). RppH prefers three or more nucleotides and the difference between three and four or more nucleotides was negligible. Therefore, we measured the activity of RppH using three nucleotides. The GCA sequence represents the first three nucleotides of the *efp* transcript, which is a known substrate of RppH (16). Upon incubation with purified wild-type RppH, pppGpCpA was converted to a monophosphorylated form, whereas the purified RppH(E56&57A) protein, in which the 56th and 57th glutamates of RppH were mutated to alanine, could not remove pyrophosphate from the substrate (Figure 3A and Supplementary Figure S6). When incubated with the Terminator^TM^ 5′-monophosphate-dependent exonuclease, the reaction product, but not the substrate, was degraded (Supplementary Figure S7). This result confirms that the 5′-monophosphorylated form is the reaction product. Notably, most *E. coli* gene transcripts downregulated by RppH begin with G or A (16). When we tested the pyrophosphohydrolase activity of RppH on pppApCpG, the same results were obtained (Figure 3B). As for the Ap_5_A hydrolase activity, DapF stimulated mRNA pyrophosphohydrolase activity of RppH, irrespective of the first nucleotide (Figure 3). The observed activation of RppH activity is unlikely to be due to a carrier effect of DapF, since the same amount of bovine serum albumin (BSA), a known carrier protein, did not activate the pyrophosphohydrolase activity of RppH. Notably, RppH was slightly more active on the triphosphorylated RNA starting with A rather than G (compare Figure 3A with B); this observation is consistent with the results from recent studies on RppH in *B. subtilis* (39,40).

**Figure 4.**
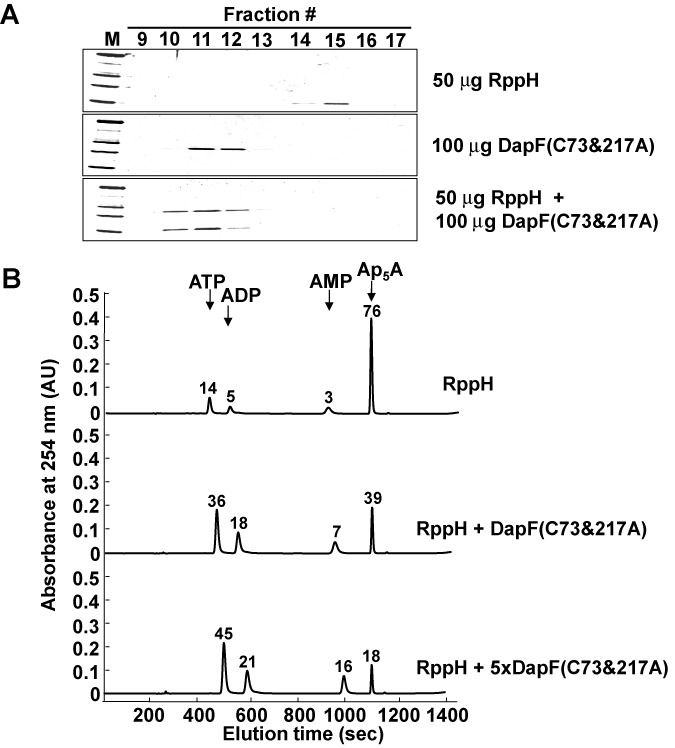
Complex formation with RppH and the activation of RppH are independent of the DAP epimerase activity of DapF. (**A**) Formation of a complex between RppH and DapF(C73&217A). Samples containing 50 μg of RppH, 100 μg of DapF(C73&217A) or both were injected onto a Superose 12 10/300 GL column, and each fraction (20 μl) was electrophoresed in SDS-PAGE gels and stained with Coomassie Brilliant Blue R. (**B**) Stimulation of RppH activity by DapF(C73&217A) in a dose-dependent manner. The Ap_5_A-hydrolysis activity of RppH (0.5 μg) was compared in the presence and absence of DapF(C73&217A) (1.5 μg or 7.5 μg). The reactions were monitored by HPLC as described in the legend to Figure 3. Relative peak areas are shown on the top of each peak.

The DapF enzyme catalyzes the conversion of L,L-DAP to *meso*-DAP, which is an intermediate for the biosynthesis of lysine and peptidoglycan (21,41). To study whether RppH activation by DapF is dependent on DapF enzymatic activity, the two catalytic cysteine residues of DapF, C73 and C217, were substituted to alanine (DapF(C73&217A)). Although the mutant protein did not catalyze the conversion of L,L-DAP to *meso*-DAP (42,43), it formed a tight complex with RppH and stimulated RppH activity (Figure 4). Because purified DapF(C73&217A) lacks the Ap_5_A hydrolase activity (Figure 2), the apparent activation of RppH by DapF(C73&217A) is unlikely to be due to contamination with RppH during the preparation of this protein. Additionally, the data in Figure 4B showed that the stimulatory effect of DapF is concentration-dependent. Therefore, these data imply that the RppH activity is regulated by the level of DapF, not the epimerase activity.

**Figure 5.**
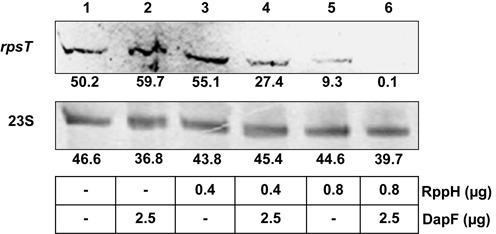
Effect of DapF on RppH-mediated conversion of the *rpsT* transcript from the triphosphate to monophosphate form. The total RNA isolated from *E. coli* was incubated with purified RppH, DapF or both and digested with the Terminator 5′-phosphate-dependent exonuclease. The remaining transcript was detected by northern blot analysis using an *rpsT*‐specific probe or a 23S rRNA-specific probe: lane 1, no addition; lane 2, 2.5 μg of DapF; lane 3, 0.4 μg of RppH; lane 4, 0.4 μg of RppH and 2.5 μg of DapF; lane 5, 0.8 μg of RppH; lane 6, 0.8 μg of RppH and 2.5 μg of DapF. Band intensities of the *rpsT* transcript were analyzed using the Multi Gauge V3.0 software and given below each lane.

To test the pyrophosphohydrolase activity of RppH on an *in vivo* substrate and to confirm the stimulatory effect of DapF on RppH activity, total RNA was extracted from wild-type cells grown in LB medium to the exponential phase. The extracted RNA was incubated with various concentrations of purified RppH, DapF or both, and each reaction mixture was treated with the Terminator 5′-phosphate-dependent exonuclease. By northern blot analysis using a specific probe, we measured the level of 5′-triphosphorylated *rpsT* P1 mRNA, which is a known substrate of RppH (16). RppH alone decreased the amount of 5′-triphosphorylated *rpsT* P1 mRNA in a concentration-dependent manner (compare lanes 1, 3 and 5 in Figure 5). Whereas DapF alone did not alter the transcript level, it significantly stimulated the RppH-mediated decay of the *rpsT* P1 transcript. The stimulation of the RppH-catalyzed pyrophosphate removal from the same transcript by DapF was further confirmed using different concentrations of RppH and DapF by both northern blot experiment and qRT-PCR (Supplementary Figure S8).

### DapF forms a tight complex with RppH and potentiates RppH activity *in vivo*

The *in vivo* interaction between DapF and RppH was tested using two methods. First, the pDuet-RD vector was constructed from the pET-duet vector to co-express DapF and His-RppH for pull-down assays. When the crude extract prepared from *E. coli* ER2566 cells harboring pDuet-RD was loaded onto the TALON^TM^ metal affinity column, DapF co-eluted with His-RppH; however, DapF was barely detectable in the eluate when the crude extract prepared from the cells overexpressing DapF alone was loaded (Supplementary Figure S9A). Based on the band intensity on the SDS-PAGE gel, RppH might interact with DapF to form a complex with a 1:1 molar ratio, which is consistent with the gel filtration chromatography data showing that DapF and RppH can form a heterotetramer with a 2:2 stoichiometry (Figure 1C).

The *in vivo* interaction between DapF and RppH was also confirmed using BACTH assays. For the BACTH assays, DapF and RppH were fused to the C-terminal ends of the T18 and T25 fragments of *B. pertussis* adenylyl cyclase, respectively. Whereas cells co-expressing the unfused T25- and T18-fragments did not develop a color, cells co-producing T18-DapF and T25-RppH developed a blue color similar to the positive control strain expressing the T25- and T18-fragments fused to the leucine zipper of the transcription factor GCN4 (Supplementary Figure S9B). Therefore, these results demonstrate that the tight interaction between RppH and DapF occurs *in vivo*.

**Figure 6.**
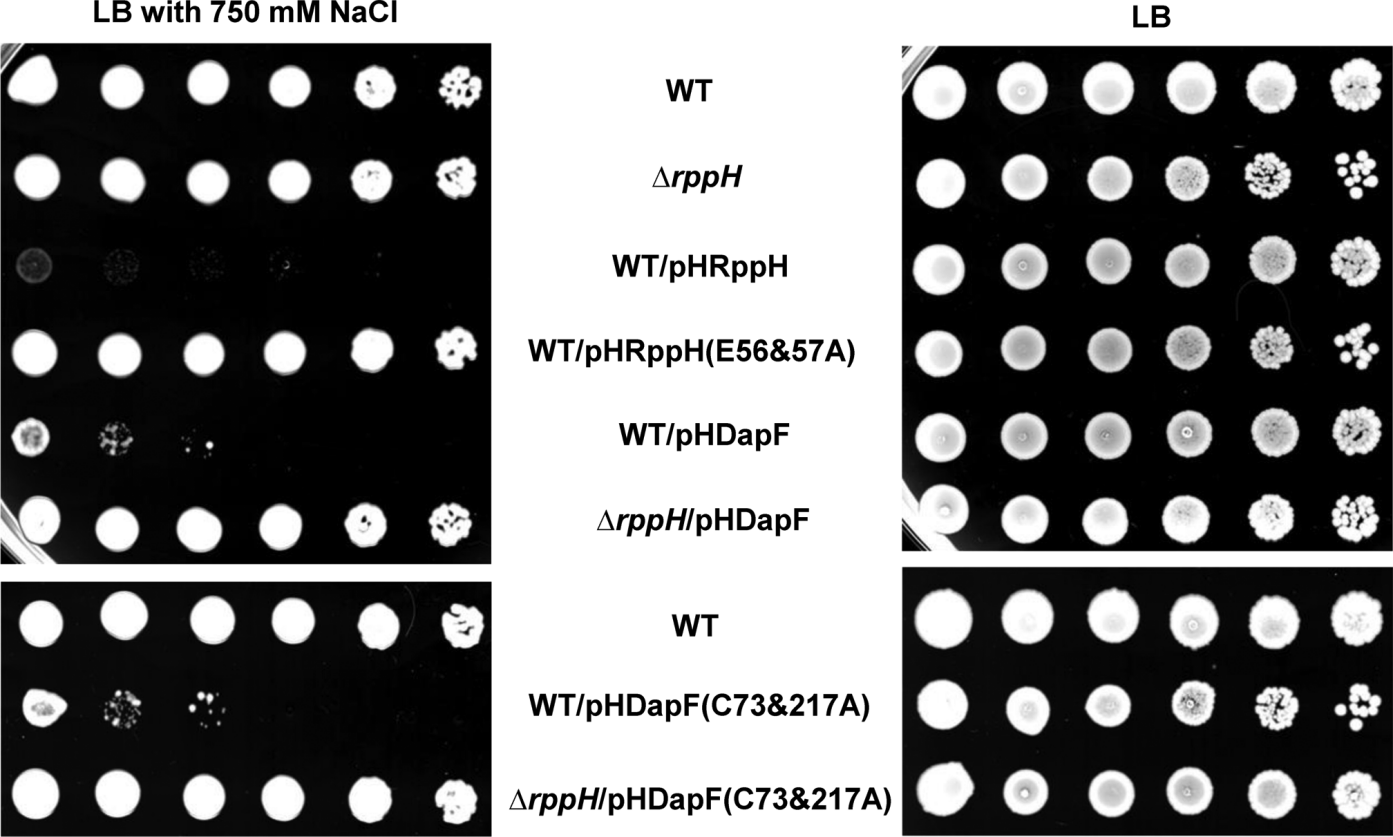
Hypersensitivity of strains with increased RppH activity to high osmolality. Stationary phase cells of the indicated strains grown in LB medium were serially diluted 10-fold from ∼10^9^ to ∼10^4^ cells/ml, and 1-μl aliquots were spotted onto LB agar plates with (left) and without (right) the addition of 750 mM NaCl. After incubation at 37°C for 16–18 h, the plates were scanned.

In bacteria, concentrations of diadenosine oligophosphates increase more than 100-fold over the endogenous level in response to heat or oxidative stress (44). Furthermore, RppH levels decrease during cellular stress (45), suggesting that RppH might be involved in stress responses. To determine the phenotype associated with RppH activity, we constructed an *rppH* mutant and strains overproducing the wild-type and mutant forms of RppH and tested the response of these strains to various stresses, including thermal, oxidative and osmotic stresses. Cells with different expression levels of RppH did not show any significant difference in growth compared to the wild type when grown in LB medium, irrespective of growth temperature or the addition of 0.2 mM H_2_O_2_. However, an RppH-overproducing strain had a significant and reproducible difference in sensitivity to osmotic stress compared to the wild-type strain. Notably, the overexpression of RppH caused hypersensitivity to high osmolality (750 mM NaCl), whereas the *rppH* mutant and the strain overproducing an inactive form of RppH (E56&57A) were as resistant as the wild-type strain (Figure 6). The expression level of mutant RppH was similar to that of the wild-type protein (Supplementary Figure S10). The *rppH* mutant with the RppH-expressing plasmid was slightly more sensitive than the wild-type and the *rppH* mutant strain, but more resistant than the wild-type strain harboring the RppH-expressing plasmid to salt stress (Supplementary Figure S11). Therefore, these data suggest that the hypersensitivity of the RppH-overproducing strain to osmotic stress is primarily due to increased enzyme activity.

Considering that DapF stimulates the activity of RppH by direct interaction, we proposed that increased expression of DapF might affect osmotic sensitivity. Overexpression of DapF in a wild-type strain led to hypersensitivity to high osmolality, whereas in the *rppH* mutant strain, the overexpression of DapF had little effect on osmotic sensitivity. To exclude the possibility that the phenotype of DapF overproduction is due to increased DAP epimerase activity, we performed the same experiment using a strain overproducing DapF(C73&217A), which has no catalytic activity but is still capable of interacting with and stimulating RppH (Figure 4). Wild-type cells overproducing the mutant DapF were as sensitive to high osmolality as the cells overproducing wild-type DapF, but the *rppH* mutant strain overproducing the mutant DapF did not show osmotic sensitivity (Figure 6). Together, these data suggest that higher RppH activity renders cells more sensitive to osmotic stress, and the effect of DapF overexpression on osmotic sensitivity is exerted through the stimulation of RppH activity.

### Stimulation of RppH pyrophosphohydrolase activity by DapF *in vivo*

**Figure 7.**
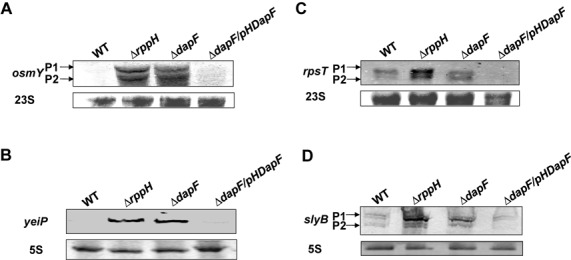
Influence of DapF on the levels of RppH substrate mRNAs. The indicated strains were grown in LB medium at 37°C. The total RNA was prepared from each strain at exponential phase and analyzed by northern blotting using standard conditions to analyze the levels of some RppH target mRNAs: (**A**) *osmY*, (**B**) *yeiP*, (**C**) *rpsT* and (**D**) *slyB*. The DIG-labeled hybridization probes are shown to the left of each panel. The blot with the 23S rRNA probe ((A) and (C)) or 5S rRNA probe ((B) and (D)) was used as a loading control.

Using gene array analysis, the abundance of 382 gene transcripts including the *rpsT* mRNA was found to be significantly higher in cells containing the mutant RppH compared to the wild-type strain (16). To validate the *in vivo* effect of DapF on RppH, concentrations of specific substrate mRNAs were measured in the wild-type, *rppH* mutant and *dapF* mutant strains by northern blot analysis. Consistent with the previous study (16), the levels of all tested substrate mRNAs were significantly higher in the *rppH* mutant cells compared to wild-type cells (Figure 7). Notably, the concentrations of the *osmY* and *yeiP* transcripts increased in the *dapF* mutant cells to levels similar to those in the *rppH* mutant cells. The concentrations of the *slyB* and *rpsT* transcripts in the *dapF* mutant cells were lower than those in the *rppH* mutant cells but still higher than those in wild-type cells. The concentrations of two *rpsT* transcripts and the *slyB* P2 transcript significantly decreased in the *dapF* mutant cells harboring pDapF, the expression vector for DapF. All tested transcripts, except the *slyB* P1 transcript, were barely detectable in the *dapF* mutant cells harboring pDapF (Figure 7). These results suggest that DapF stimulates the pyrophosphohydrolase activity of RppH *in vivo*.

**Figure 8.**
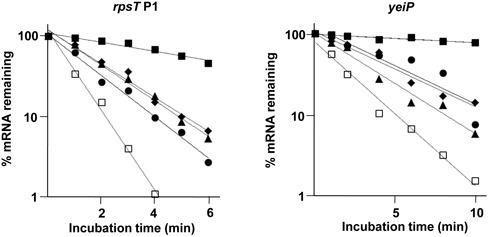
Effect of DapF on the decay rates of RppH target mRNAs. The total RNAs were extracted from the wild-type (closed diamonds), *rppH* mutant (closed squares), RppH-overexpressing (closed triangles) and DapF(C73&217A)-overexpressing (closed circles) strains, and the strain overexpressing both RppH and DapF(C73&217A) (open squares) at the indicated times after inhibiting transcription by the addition of rifampin. Transcript levels were analyzed by qRT-PCR with primers specific for *rpsT* P1, *yeiP* or 16S rRNA. The mRNA levels were normalized to the concentration of 16S rRNA and plotted as a function of time. Average data from two independent experiments are shown.

Next, we tested whether the rate of mRNA decay is sensitive to increasing the concentration of RppH or DapF. Cells were grown to the exponential phase, and the total cellular mRNAs were extracted at several time intervals after the addition of rifampin to block transcription. The rate of mRNA decay was analyzed by qRT-PCR with primers specific for known RppH targets. An increase in the intracellular concentration of RppH alone had only a slight, if any, effect on the decay rates of these transcripts (Supplementary Figure S12). This result is consistent with the previous report demonstrating that the overexpression of RppH does not influence the lifetimes of some RppH targets (33). However, the overexpression of DapF(C73&217A) slightly increased the decay rates of some transcripts (*rpsT* P1, *osmY* and *slyB*), whereas other transcripts (*ydfG*, *yfcZ* and *yeiP*) were not affected by the overexpression of DapF(C73&217A). Although the half-life of the *yeiP* transcript was not influenced by the overexpression of DapF(C73&217A) alone, the half-life of the *yeiP* transcript was considerably shortened in cells overexpressing both RppH and DapF(C73&217A), like that of the *rpsT* P1 transcript (Figure 8). Similar to results from northern blot analyses, the decay rates of all tested transcripts significantly decreased in the *dapF* mutant as well as in the *rppH* mutant (Figure 8 and Supplementary Figure S13). Therefore, these results support the hypothesis that DapF is an ancillary factor stimulating the pyrophosphohydrolase activity of RppH *in vivo*.

**The salt sensitivity of cells with an increased RppH activity is suppressed by overexpression of the *osmY* gene**

The overproduction of wild-type but not mutant RppH renders cells extremely sensitive to high salt, and cells overproducing DapF (irrespective of its catalytic activity) have salt sensitivity similar to cells overproducing wild-type RppH (Figure 6). These results suggest that one or more RppH target messages are required during growth at high osmolality and that DapF potentiates RppH-catalyzed conversion of their triphosphorylated ends to monophosphates. Among known targets of RppH (16), *osmY* encodes a periplasmic protein whose expression is inducible under hyperosmotic conditions, and mutation of the *osmY* gene increases sensitivity to hyperosmotic stress (46). Notably, our data show that the *omsY* transcript level in the *dapF* mutant is similar to that in the *rppH* mutant cells and much higher compared to the level in wild-type cells (Figure 7). Furthermore, the decay rate of the *osmY* transcript was slightly increased by the overexpression of DapF(C73&217A) and significantly decreased by the *dapF* deletion (Supplementary Figures S12 and S13). We therefore constructed an *osmY* mutant and an *osmY* expression vector to explore the possibility that the hyperosmotic sensitivity of cells overproducing RppH and DapF may be due to the decreased expression level of *osmY*.

**Figure 9.**
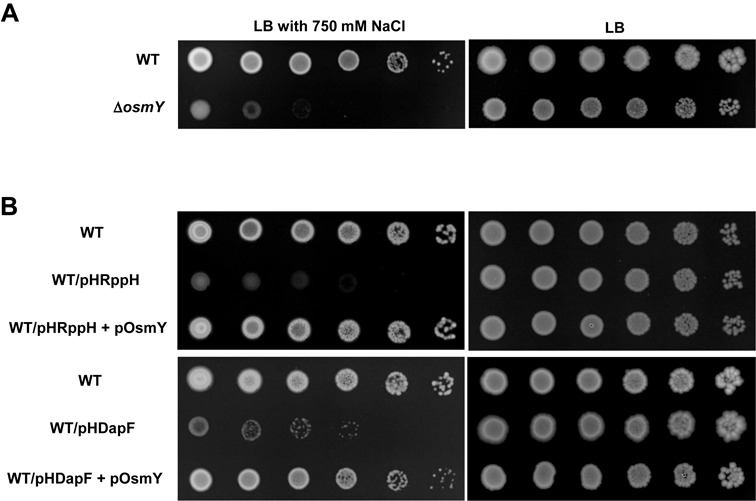
Salt hypersensitivity of cells with an increased RppH activity is suppressed by overexpression of the *osmY* gene. Effects of *osmY* deletion (**A**) and overexpression (**B**) on the salt sensitivity of the wild-type strain (WT), and strains harboring RppH and DapF expression vectors were measured. Stationary phase cells of the indicated strains grown in LB medium were serially diluted 10-fold from ∼10^9^ to ∼10^4^ cells/ml, and 1-μl aliquots were spotted onto LB agar plates with (left) and without (right) the addition of 750 mM NaCl. Isopropyl-β-D-thiogalactopyranoside (IPTG) was added to 1 mM to induce expression of OsmY in (B). After incubation at 37°C for 16–18 h, the plates were scanned.

Consistent with the previous study (46), the *osmY* mutant was as sensitive to hyperosmotic stress as the strain overproducing RppH or DapF (Figure 9). In addition, the salt hypersensitivity of the RppH- and DapF-overproducing strains was suppressed by overexpression of the *osmY* gene. Although further studies are required to fully understand the relationship between RppH-mediated mRNA degradation and the hyperosmotic stress response, our data suggest that the salt sensitivity of RppH- and DapF-overexpressing strains is likely to be due to the decreased level of the *osmY* transcript. The decrease in the *osmY* mRNA level could be due, at least in part, to the increased pyrophosphohydrolase activity. In this regard, the significantly decreased expression level of DapF in stationary phase cells compared to exponentially growing cells (Supplementary Figure S14A) might explain the previous result showing that expression of the *osmY* gene is induced upon entry into stationary phase (47). It should be noted that the RppH level remained relatively constant regardless of growth phase. Therefore, our data suggest that the cellular level of DapF is a critical factor regulating the RppH activity.

## DISCUSSION

In parallel with the mechanism of mRNA decay in eukaryotic organisms, the status of the 5′ end is critical to mRNA decay in bacteria (48). In *E. coli*, the RNA pyrophosphohydrolase RppH initiates mRNA decay by converting the triphosphorylated terminus to a monophosphorylated form, which can accelerate internal mRNA cleavage by RNase E (16). Despite the physiological importance of RppH-mediated mRNA decay, the regulation of RppH activity has not been studied. A recent study suggested the existence of an ancillary factor for RppH by showing that the overproduction of RppH alone did not increase the 5′-monophosphorylated products of some target mRNAs (33). In this study, we identified the diaminopimelate epimerase DapF as a regulator of RppH-mediated mRNA degradation in *E. coli*. DapF stimulated the mRNA pyrophosphohydrolase activity of RppH by direct protein–protein interaction with unusually high affinity (*K*_d_ ∼ 5.2 × 10^−9^ M) and this stimulation was independent of the catalytic activity of DapF (Figures 2–4). Whereas increased expression of RppH alone had only a slight effect on the decay rates of the tested mRNAs, the coexpression of RppH with DapF significantly increased the decay rates of these transcripts. Furthermore, several transcripts were stabilized in the *dapF* mutant and in the *rppH* mutant and the overproduction of DapF accelerated the decay of some of these transcripts (Figure 7), suggesting that the cellular level of DapF is a critical factor regulating the RppH-catalyzed pyrophosphate removal and subsequent degradation of target mRNAs.

Together with previous reports, our data suggest that the major physiological role of DapF might be to stimulate RppH activity rather than to produce *meso*-diaminopimelate (DAP). *meso*-DAP is an essential component of the cell wall peptidoglycan and is decarboxylated by LysA to generate L-lysine in most Gram-negative bacteria. *meso*-DAP can be synthesized by two pathways in bacteria: the epimerase pathway in which DapF converts L,L-DAP to *meso*-DAP and the dehydrogenase pathway in which DAP dehydrogenase directly converts tetrahydrodipicolinate to *meso*-DAP (37). However, because the dehydrogenase pathway exists only in a small number of Gram-positive bacteria such as *Bacillus sphaericus* (37), *meso*-DAP and consequently L-lysine are thought to be generated only through the epimerase pathway in *E. coli*. However, a *dapF* deletion mutant did not require *meso*-DAP or L-lysine for growth and could synthesize *meso*-DAP, although this mutant accumulated large amounts of L,L-DAP, the substrate of the epimerase reaction (21,41). These data suggest that there might be another enzyme capable of catalyzing the DAP epimerase reaction at a rate sufficient to sustain growth in *E. coli*.

If the synthesis of *meso*-DAP from L,L-DAP is the major role of DapF, the expression level of DapF should increase in cells grown in a medium deficient in L-lysine compared to cells grown in a medium supplemented with the amino acid. However, the expression level of DapF was higher in *E. coli* cells grown in lysine-containing media (LB and M9 supplemented with casamino acids) compared to cells grown in M9 medium without amino acids (Supplementary Figure S14B). Furthermore, many of the currently identified mRNA targets of RppH are responsible for the synthesis of amino acids such as His, Trp, Tyr, Phe, Thr, Ile and Val (16). Therefore, the expression level of DapF might increase in amino acid-rich medium to stimulate the decay of mRNAs involved in the biosynthesis of amino acids. Further studies are required to elucidate the mechanism regulating DapF expression level and to delineate the connection between RppH-mediated mRNA degradation and amino acid metabolism.

Enolase is a glycolytic enzyme that catalyzes the dehydration of 2-phospho-D-glycerate to form phosphoenolpyruvate and the reverse reaction in gluconeogenesis (49). In *E. coli*, approximately one-tenth of the total enolase is associated with RNase E, the polynucleotide phosphorylase PNPase and the ATP-dependent RNA helicase RhlB to form the RNA degradosome (50). The role of enolase in the degradosome has not been definitely established. However, mutational analyses demonstrated that enolase within the degradosome plays a crucial role in the regulation of *ptsG* mRNA stability in the response to phosphosugar stress (51), which is mediated by the small regulatory RNA SgrS. Another study using DNA microarray analyses suggested that the association of enolase with RNase E in the degradosome affects transcripts that encode enzymes of energy-generating pathways (52). In this study, we provide another instance where a metabolic enzyme is involved in an RNA degradation complex. Like enolase, DapF might specifically target genes involved in certain metabolic pathways such as the biosynthesis of amino acids. Further studies on the regulation of DapF expression and the effect of DapF on the affinity of RppH for their target mRNAs may help to understand why some mRNAs respond differently to RppH and DapF levels.

## SUPPLEMENTARY DATA

Supplementary Data are available at NAR Online.

SUPPLEMENTARY DATA
