# Supplementary material for: RppH-dependent pyrophosphohydrolysis of mRNAs is regulated by direct interaction with DapF in Escherichia coli
Source: Nucleic Acids Res. 2014 Oct 13;42(20):12746–57. doi: 10.1093/nar/gku926 (PMC4227774; doi:10.1093/nar/gku926)
Supplement: SUPPLEMENTARY DATA [file supp_42_20_12746__index.html]

RppH-dependent pyrophosphohydrolysis of mRNAs is regulated by direct interaction with DapF in Escherichia coli — RppH-dependent pyrophosphohydrolysis of mRNAs is regulated by direct interaction with DapF in Escherichia coli — SUPPLEMENTARY DATA 

# RppH-dependent pyrophosphohydrolysis of mRNAs is regulated by direct interaction with DapF in *Escherichia coli*

## SUPPLEMENTARY DATA

**Files in this Data Supplement:**

- SUPPLEMENTARY DATA
