# Supplementary material for: RppH-dependent pyrophosphohydrolysis of mRNAs is regulated by direct interaction with DapF in Escherichia coli
Source: Nucleic Acids Res. 2014 Oct 13;42(20):12746–57. doi: 10.1093/nar/gku926 (PMC4227774; doi:10.1093/nar/gku926)
Supplement: SUPPLEMENTARY DATA [file supp_gku926_nar-01280-y-2014-File003.pdf]

Supplementary data

Table S1. *Escherichia coli* strains and plasmids used in this study.

Strain or plasmid	Genotype or phenotype	Source or Reference
**Strains**		
MG1655	F^- λ^- *ilvG*$^-$ *rfb-50 rph-1*. Wild type *E. coli* K-12	(1)
GI698	F^- λ^- *lacI*^{q} *lacPL8 ampC*::P_{trp} cI	(2)
DY330	W3110 Δ*lacU169 gal490* λ*CI857* Δ*(cro-bioA)*	(3)
KM100	DY330 *rppH::*Km^R	This study
KM200	DY330 *dapF::*Tet^R	This study
KM101	MG1655 *rppH::*Km^R	This study
KM201	MG1655 *dapF::*Tet^R	This study
KM301	MG1655 *osmY::*Tet^R	This study
**Plasmids**		
pRE1	Expression vector under control of λP_L promoter, Amp^r	(4)
pRppH	pRE1-based expression vector for RppH	This study
pHRppH	pRE1-based expression vector for RppH with N-terminal 6 histidines	(5)
pDapF	pRE1-based expression vector for DapF	This study
pHDapF	pRE1-based expression vector for DapF with N-terminal 6 histidines	This study
pHRppH(E56&57A)	pRE1-based expression vector for RppH(E56&57A) with N-terminal 6 histidines	This study
pHDapF(C73&215A)	pRE1-based expression vector for DapF(C73&215A) with N-terminal 6 histidines	This study
pCR3H	pRE1-based expression vector for $EIIA^{Ntr}$ with N-terminal 6 histidines	(6)
pETDuet-1		Novagen
pDuet-D	pETDuet-1-based expression vector for DapF	This study
pDuet-RD	pETDuet-1-based expression vector for His-RppH and DapF	This study
pUT18c	ColEI-ori, P_{lac}::*cyaA* 225–399, encoding *B. pertussis* CyaA T18 fragment, Amp^R	(7)
pUT18c-zip	ColEI-ori, P_{lac}::*cyaA* 225–399φGCN4-zip, Amp^R	(8)
pUT18c-RppH	Contains *E. coli* RppH fused to *B. pertussis* CyaA T18 fragment, Amp^R	This study
pKT25	ori p15A, P_{lac}::*cyaA* 1–224, encoding *B. pertussis* CyaA T25 fragment, Km^R	(7)
pKT25-zip	ori p15A, P_{lac}::*cyaA* 1–224φGCN4-zip, Km^R	(9)
pKT25-DapF	Contains *E. coli* DapF fused to *B. pertussis* CyaA T25 fragment, Km^R	This study
pET24a		Novagen
pOsmY	pET24a-based OsmY expression vector, Km^R	This study

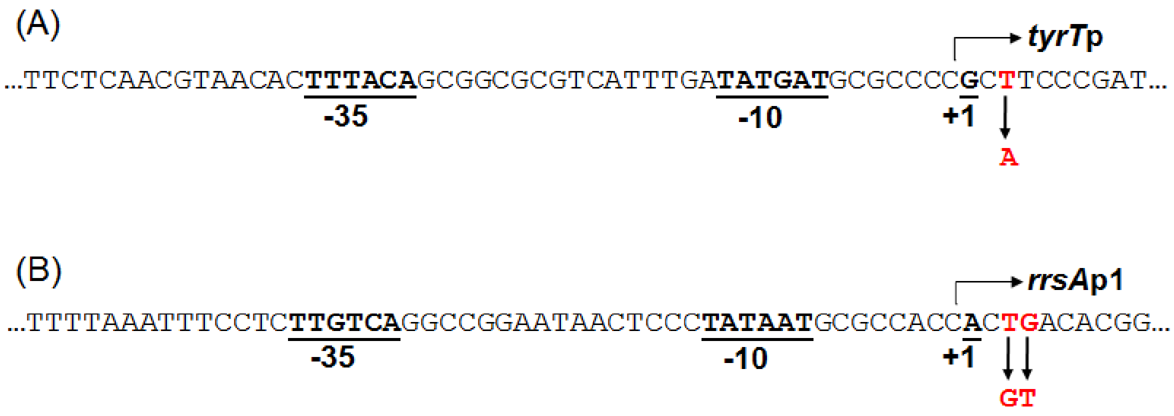

Figure S1. Linear DNA templates to synthesize pppGpCpA and pppApCpG

Small mRNA substrates to assay the pyrophosphohydrolase activity of RppH were synthesized from two strong promoters (*tyrT*p and *rrsA*p1) using the *E. coli* σ^{70}-RNAP holoenzyme. The DNA sequences near the transcription start sites of the two promoters were modified as indicated (red bases with arrows) to start transcription with GCAT (A) and ACGT (B), respectively. The regions spanning -60 to +25 relative to the transcription start sites were amplified by PCR and used as templates for transcription reactions. After 1 mM ATP, CTP, and GTP were added, the reaction mixtures were incubated at 37 °C for 2 hr. Because UTP was not added to the reaction, transcription was terminated at the third base. The −10 and −35 regions and transcription start sites (+1) are in bold face and underlined as indicated.

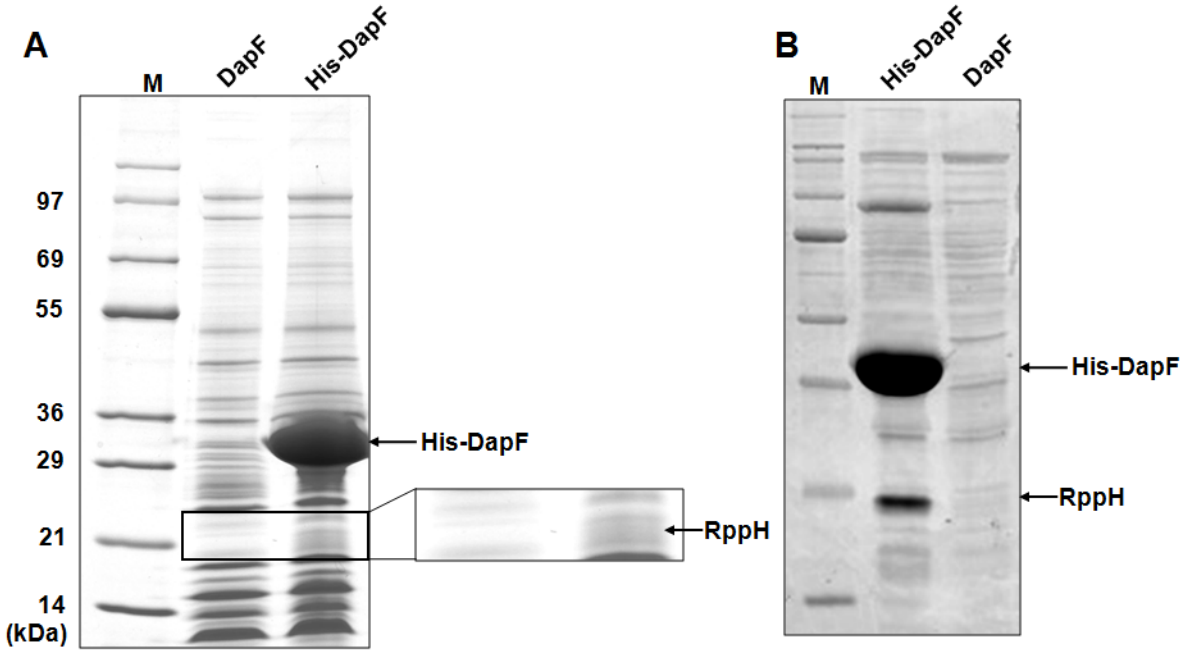

Figure S2. Ligand fishing experiments using His-DapF as bait

Crude extracts prepared from wild-type MG1655 (**A**) and RppH-overexpressing cells (**B**) grown in 500 ml of LB to stationary phase were mixed with 500 μg of purified DapF or His-DapF as indicated. Each mixture was incubated with 500 μl of TALON resin for metal affinity chromatography. After a brief wash, the proteins bound to each column were eluted with the binding buffer containing 200 mM imidazole, and the eluted samples were run on a 4-20% gradient (**A**) or 15% polyacrylamide gel (**B**). The EzWayTM Protein Blue MW Marker (KOMA Biotech) was used as the molecular mass markers (lane M).

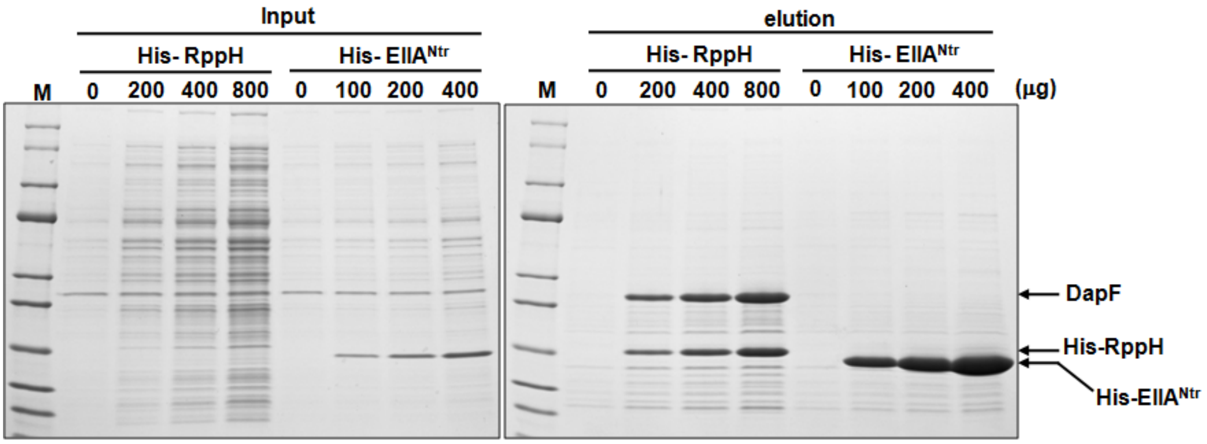

Figure S3. Specific interaction of DapF with RppH

Partially purified DapF (50 µg) was mixed with various amounts of *E. coli* cell extract expressing His-RppH or His-EIIA^{Ntr} in a total volume of 1 ml. A 10-µl aliquot was withdrawn for input control of each mixture and analyzed by 4-20% gradient SDS-PAGE and staining with Coomassie Brilliant Blue (input). The rest of each mixture was incubated with 50 µl of TALON resin in a column for 30 min at 4 °C. After each column was washed with 10 volumes of the binding buffer (50 mM Tris·HCl, pH 8.0, containing 300 mM NaCl), the bound proteins were eluted with 50 µl of 2x SDS sample buffer. Aliquots (20 µl) of the eluted samples were analyzed by SDS-PAGE and Coomassie Blue staining (elution).

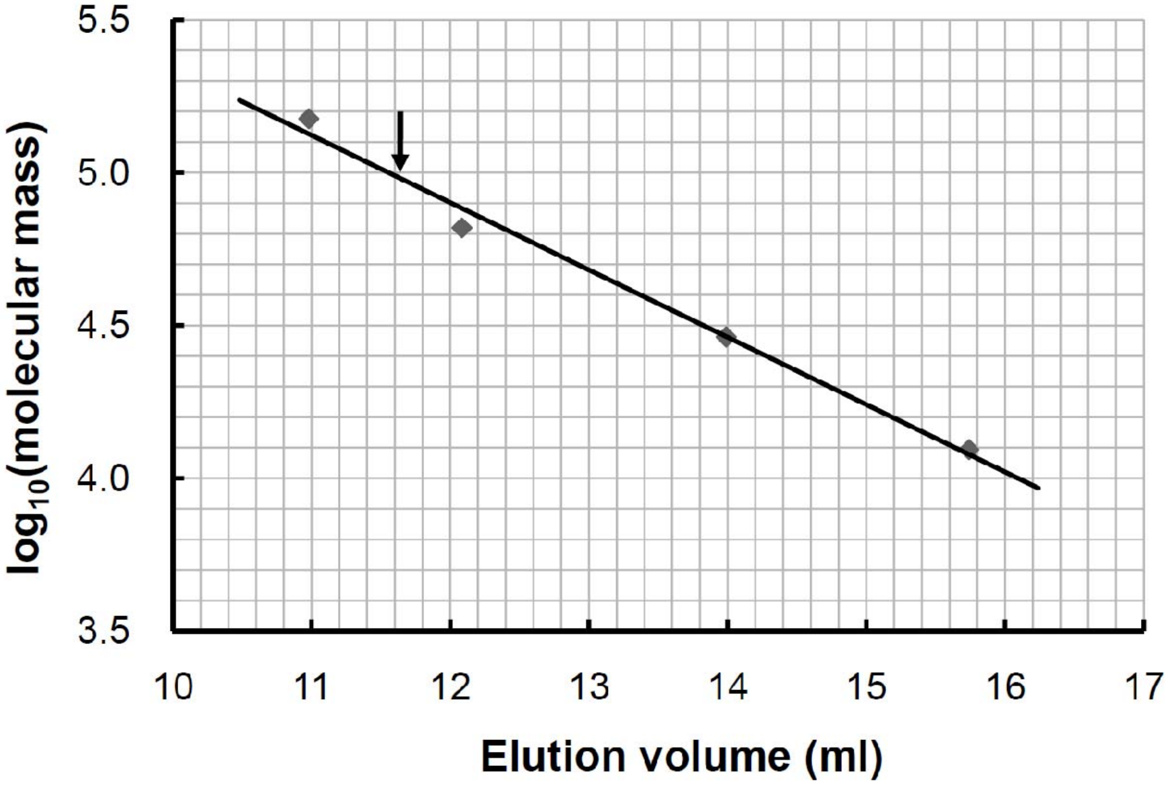

Figure S4. Determination of molecular mass of the RppH-DapF complex by gel filtration chromatography

Gel filtration chromatography was performed on a Superose 12 10/300 GL column equilibrated with 50 mM Tris-HCl (pH 8.0) containing 100 mM NaCl in an ÄKTA-FPLC system (GE Healthcare Life Sciences). Gel filtration was performed at room temperature at a flow rate of 0.5 ml/min and protein elution was monitored by measuring the absorbance at 280 nm. The column was calibrated using size markers (Sigma-Aldrich): horse heart cytochrome c (12.4 kDa), bovine carbonic anhydrase (29 kDa), bovine serum albumin (66 kDa), and yeast alcohol dehydrogenase (150 kDa). For comparison, the position of the elution peak for the RppH-DapF complex is indicated with an arrow.

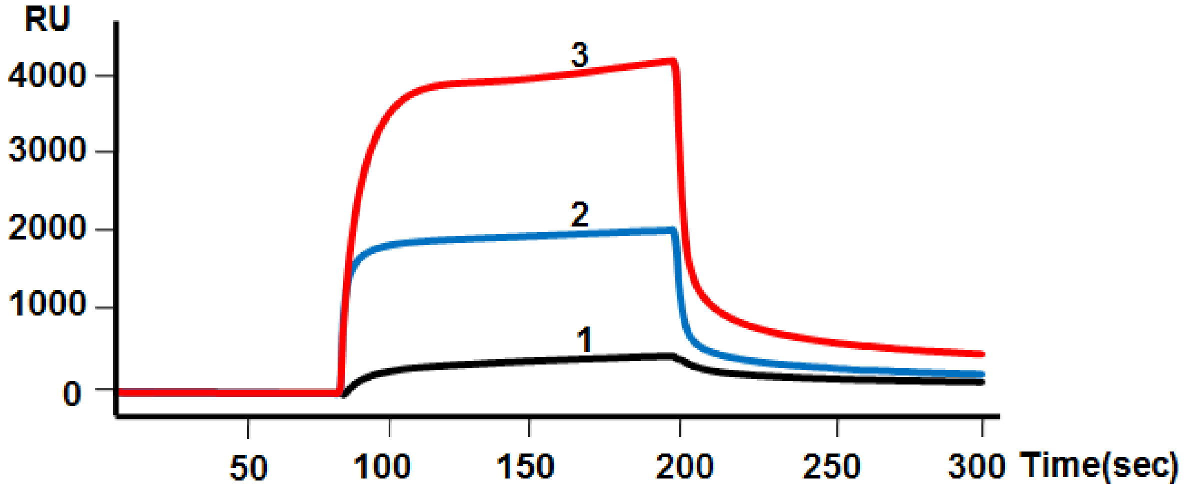

Figure S5. Kinetics of the interaction between DapF and RppH

Purified RppH was immobilized on the carboxymethylated dextran surface of a CM5 sensor chip. Three different concentrations of purified DapF were flowed over the RppH surface for 2 min in each experiment: 1, 20 μg/ml; 2, 50 μg/ml; 3, 70 μg/ml. Using BIAevaluation 2.1 software, the dissociation constant (K_d) for the interaction between RppH and DapF was determined to be approximately 5.2 x 10^{-9} M.

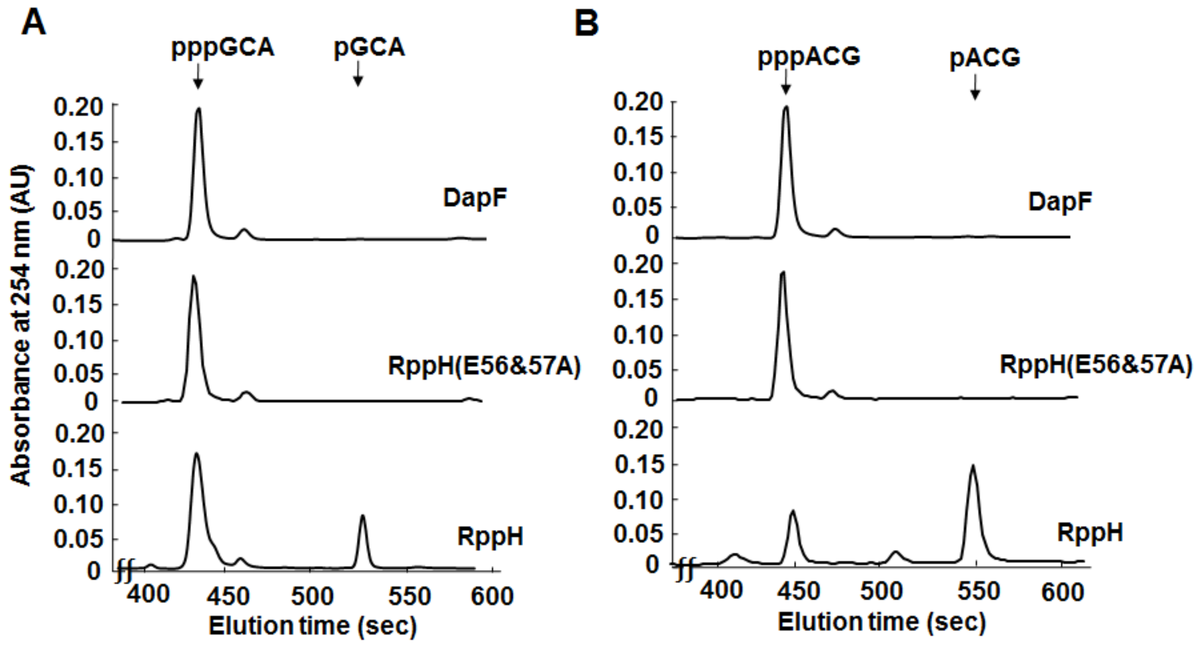

Figure S6. Purified DapF and RppH(E56&57A) do not show any pyrophosphohydrolase activities

Purified DapF (1.5 μg) and RppH(E56&57A) (0.5 μg) were incubated with two synthetic RNAs (pppGpCpA (**A**) and pppApCpG (**B**)) at 37 ºC for 10 min and their pyrophosphohydrolase activities were analyzed by reverse phase chromatography using a Varian dual pump HPLC system (see Materials and Methods). A Hypersil Gold C18 column (Thermo Scientific) was pre-equilibrated with 20 mM ammonium acetate buffer (pH 5.0) in water, and triphosphorylated and monophosphorylated RNAs were separated by using a linear gradient of 0-20% 20 mM ammonium acetate in methanol at a flow rate of 1 ml/min for 20 min. The eluted nucleotides were monitored by measuring the A_{254}. The pyrophosphohydrolase activity of wild-type RppH (0.5 μg) was included as a control. Reactions were terminated by the addition of trifluoroacetic acid to a final concentration of 2%.

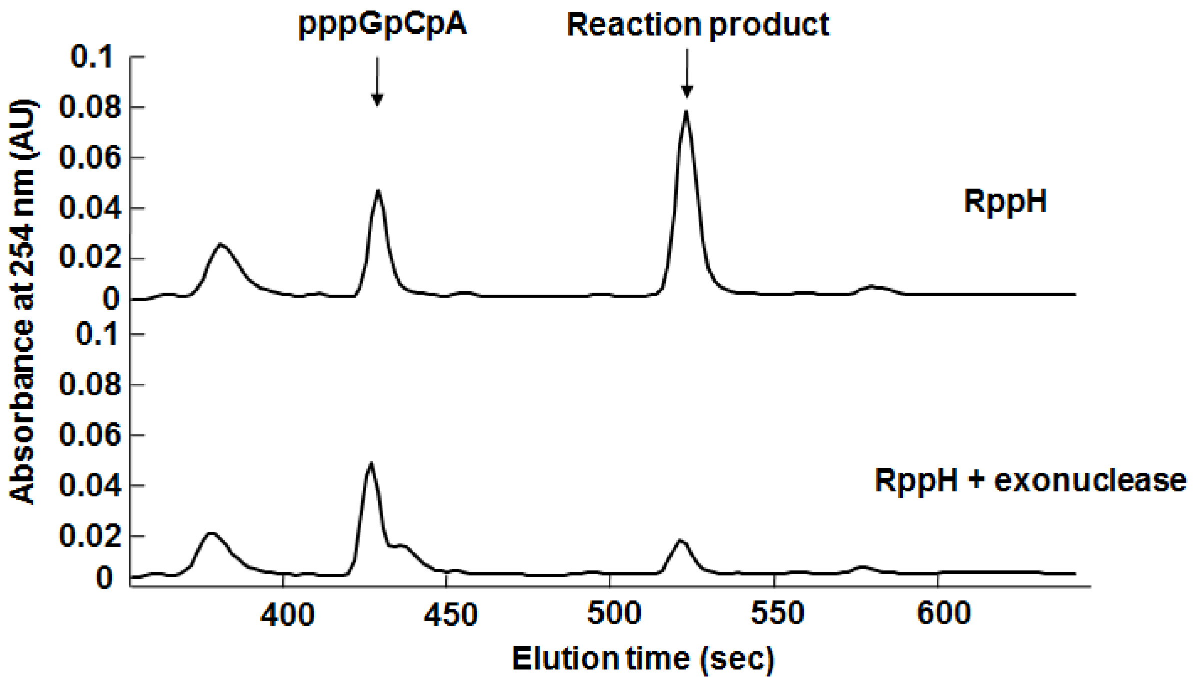

Figure S7. Verification of monophosphorylated RNA as the RppH reaction product using a 5'-monophosphate-dependent exonuclease

Activities of RppH and exonuclease on pppGpCpA synthesized by *E. coli* RNA polymerase were analyzed by HPLC (compare with Figure 3A). pppGpCpA was incubated with RppH (upper chromatogram), and after stopping the RppH reaction, the mixture was incubated with the Terminator 5′-phosphate-dependent exonuclease (lower chromatogram). Specific degradation of the product but not the substrate by the exonuclease verified that pGpCpA is the reaction product generated by RppH.

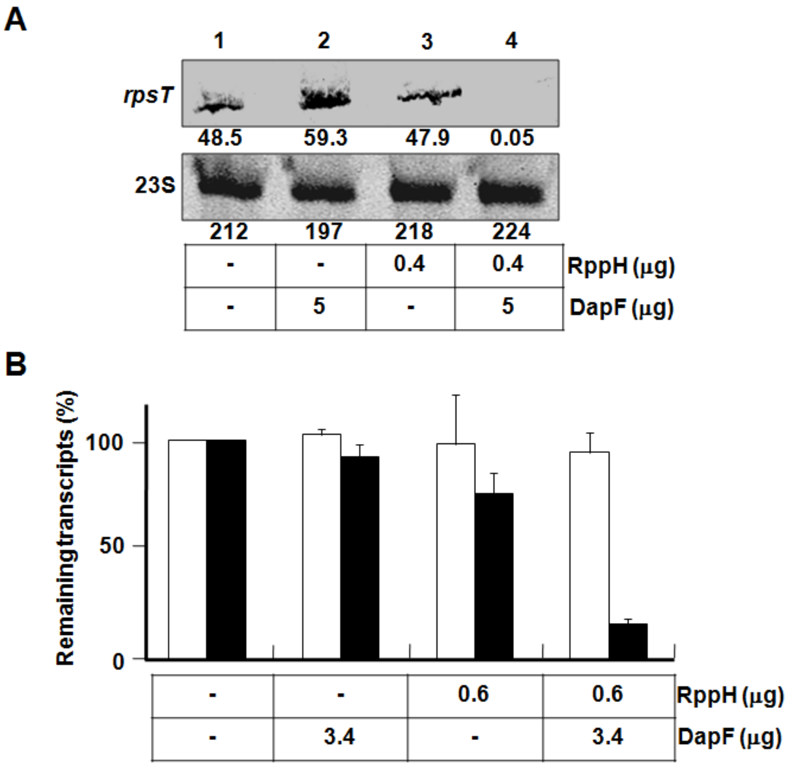

Figure S8. Effect of DapF on RppH-catalyzed conversion of the *rpsT* transcript from the triphosphate to monophosphate form

(**A**) The total RNA isolated from *E. coli* grown to an OD_{600} of 0.8 in LB medium was incubated with the indicated amounts of purified RppH, DapF or both at 37 °C for 1 hr and digested with the Terminator 5'-phosphate-dependent exonuclease. The remaining transcript was detected by Northern blot analysis using an *rpsT*-specific or a 23S rRNA-specific probe. Band intensities of the *rpsT* P1 transcript were analyzed using the Multi Gauge V3.0 software and given below each lane. (**B**) The total RNA isolated from *E. coli* was incubated with purified RppH, DapF or both at 37 °C for 1 hr in reaction buffer containing 50 mM Tris-HCl (pH 8.8) and 5 mM $MgCl_2$, and digested with the Terminator 5'-phosphate-dependent exonuclease. After phenol extraction and ethanol precipitation, the remaining transcript was analyzed by qRT-PCR using the *rpsT*-specific or 16S rRNA-specific probe: white bars, 16S rRNA; black bars, *rpsT*.

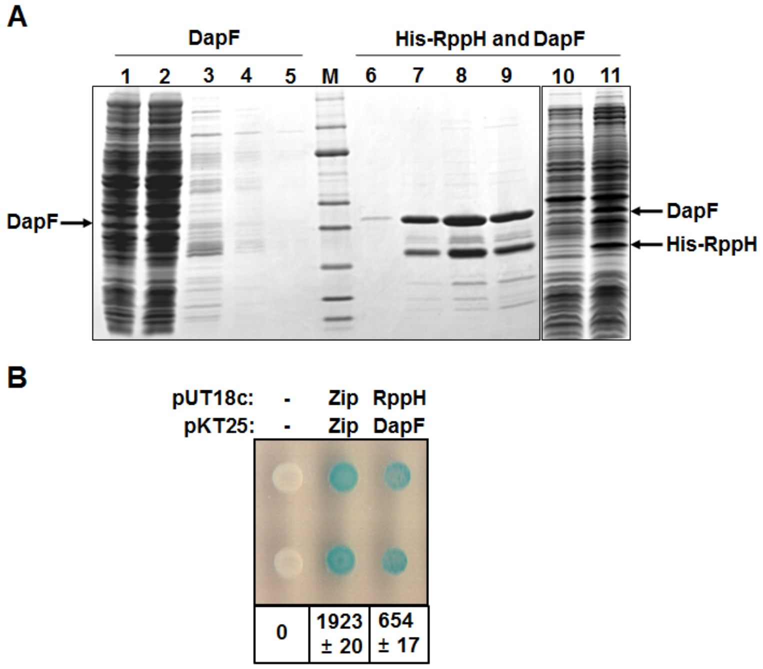

Figure S9. Specific interaction of DapF with RppH *in vivo*

(**A**) Co-purification of His_6-RppH and DapF. ER2566 cells harboring pDuet-D expressing DapF alone (lanes 1-5) or pDuet-RD co-expressing His-RppH and DapF (lanes 6-11) were grown in 100 ml of LB medium, and protein expression was induced by adding 1 mM IPTG. The cell suspension was disrupted in a French Pressure cell and centrifuged at 10,000 x *g*, and the supernatant was mixed with 200 µl of BD TALON^{TM} metal affinity resin. The column was washed three times, and the proteins bound to the column were eluted four times using 200 mM imidazole: *lane 1*, crude cell extract after induction of DapF expression; *lane 2*, clarified supernatant; *lane 3*, first eluted fraction containing DapF; *lane 4*, second eluted fraction; *lane 5*, third eluted fraction; *lane M*, molecular mass markers; *lane 6*, fourth eluted fraction containing His-RppH and DapF; *lane 7*, third eluted fraction; *lane 8*, second eluted fraction; *lane9*, first eluted fraction; *lane 10*, cells before induction; *lane 11*, cells after induction of His-RppH and DapF expression. (**B**) The BACTH system was used to analyze the interaction of RppH with DapF *in vivo*. RppH and DapF were fused to the C-terminal ends of the T18 and T25 fragments of *B. pertussis* adenylyl cyclase, respectively. Co-transformants of *E. coli* strain BTH101 expressing the indicated fusion proteins were spotted on LB plates containing 100 µg/ml streptomycin, 100 µg/ml ampicillin, and 50 µg/ml kanamycin with 40 µg/ml X-gal as the color indicator for β-galactosidase activity and incubated at 30 °C overnight. Protein-protein interaction was monitored by the β-galactosidase-mediated color development on an X-Gal plate. Transformants expressing the unfused T25- and T18-fragments served as negative controls and cells producing the T25- and T18-fragments fused to the leucine zipper of the transcription factor GCN4 were used as positive controls. Numbers below the lanes indicate β-galactosidase activities in Miller units determined in cells grown at 30 °C to OD_{600} of 0.8 in LB medium (mean ± S.D. of triplicate determinations).

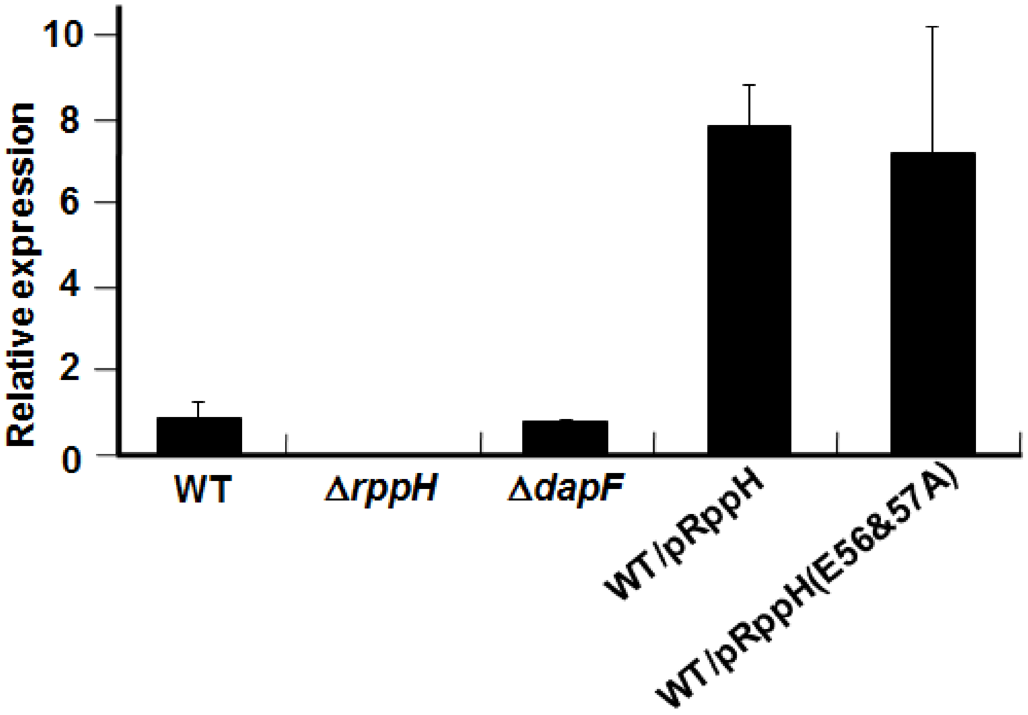

Figure S10. Expression profiles of the *rppH* transcript in various strains

The expression levels of the *rppH* transcript in the indicated strains were measured using qRT-PCR. The mRNA levels were normalized to the concentration of 16S rRNA.

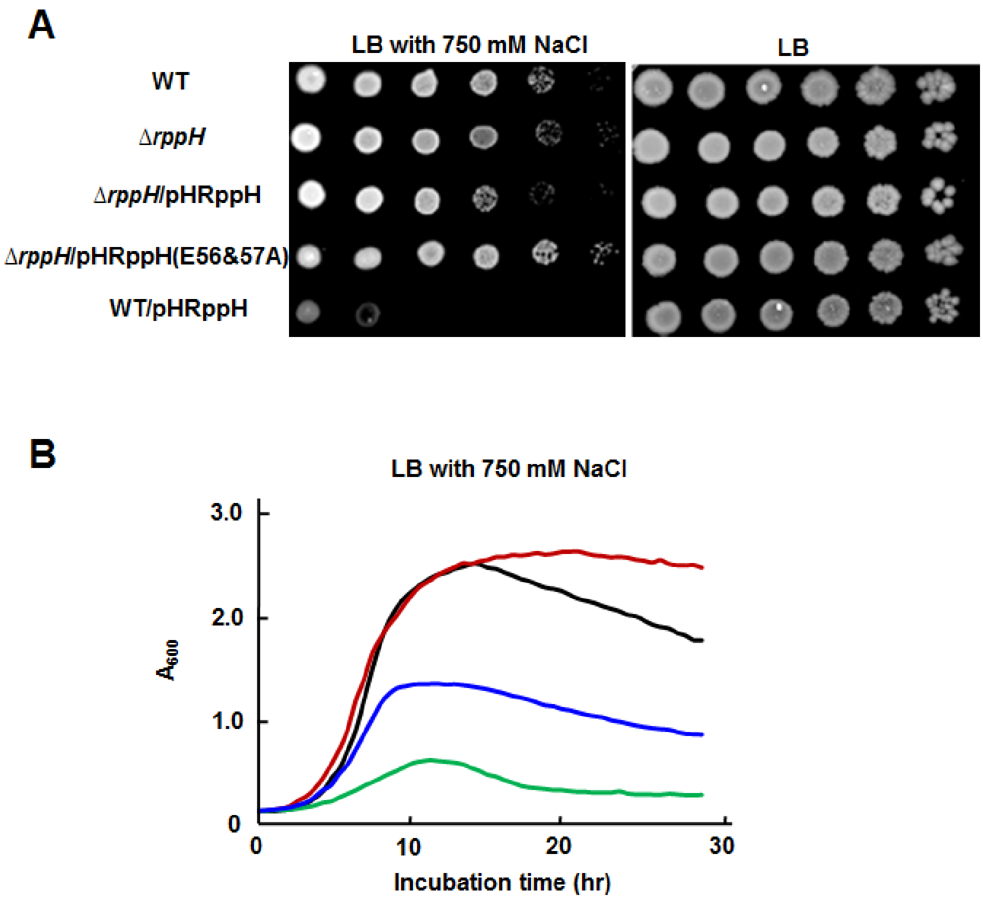

Figure S11. Hypersensitivity of strains with increased RppH activity to salt stress

(**A**) Stationary phase cells of the indicated strains grown in LB medium were serially diluted 10-fold from ~10^9 to ~10^4 cells/ml, and 1-µl aliquots were spotted onto LB agar plates with (left) and without (right) the addition of 750 mM NaCl. After incubation at 37 °C for 16–18 h, the plates were scanned. (**B**) Cells grown in LB medium overnight were inoculated into LB medium containing 750 mM NaCl, and growth at 37 °C was recorded by measuring the optical density at 600 nm: black line, MG1655; red, KM101(Δ*rppH*); blue, KM101/pRppH; and green, MG1655/pRppH.

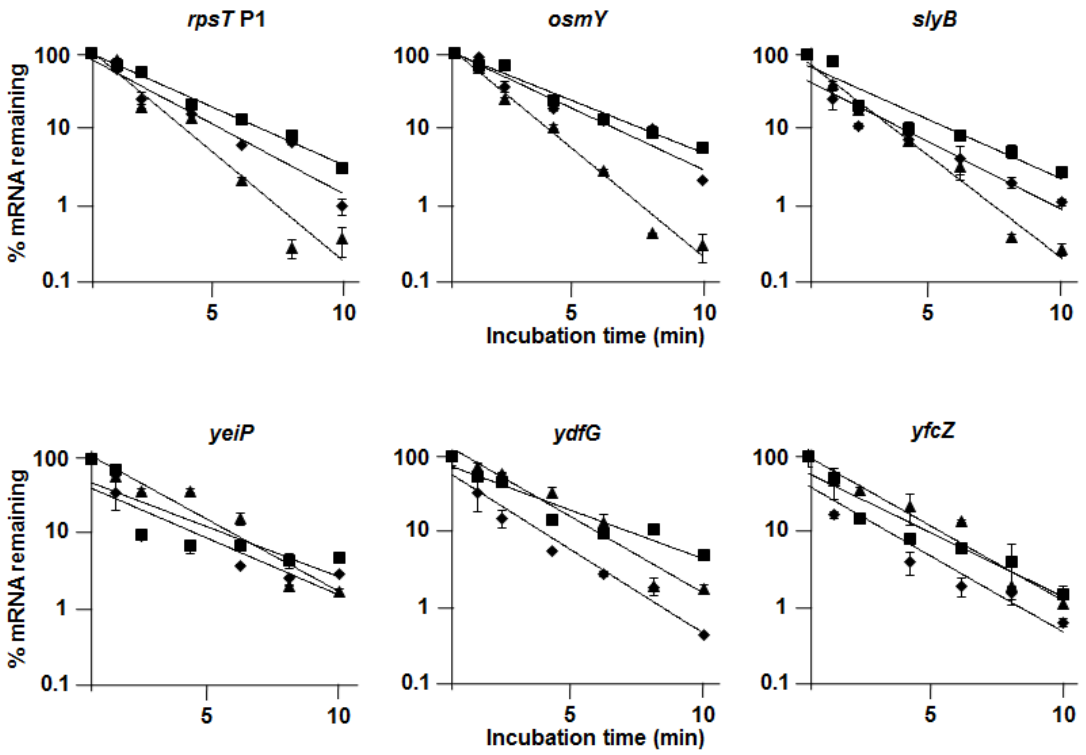

Figure S12. Effect of the overexpression of RppH and DapF(C73&217A) on the decay rates of RppH target mRNAs

The total RNAs were extracted from the wild-type (closed diamonds), RppH-overexpressing (closed squares), and DapF(C73&217A)-overexpressing (closed triangles) strains at the indicated times after inhibiting transcription by the addition of rifampin. Transcript levels were analyzed by qRT-PCR with primers specific for the *rpsT* P1, *osmY*, *slyB*, *yeiP*, *ydfG*, *yfcZ*, or 16S rRNA. The mRNA levels were normalized to the concentration of 16S rRNA and plotted as a function of time. Average data from two independent experiments are shown.

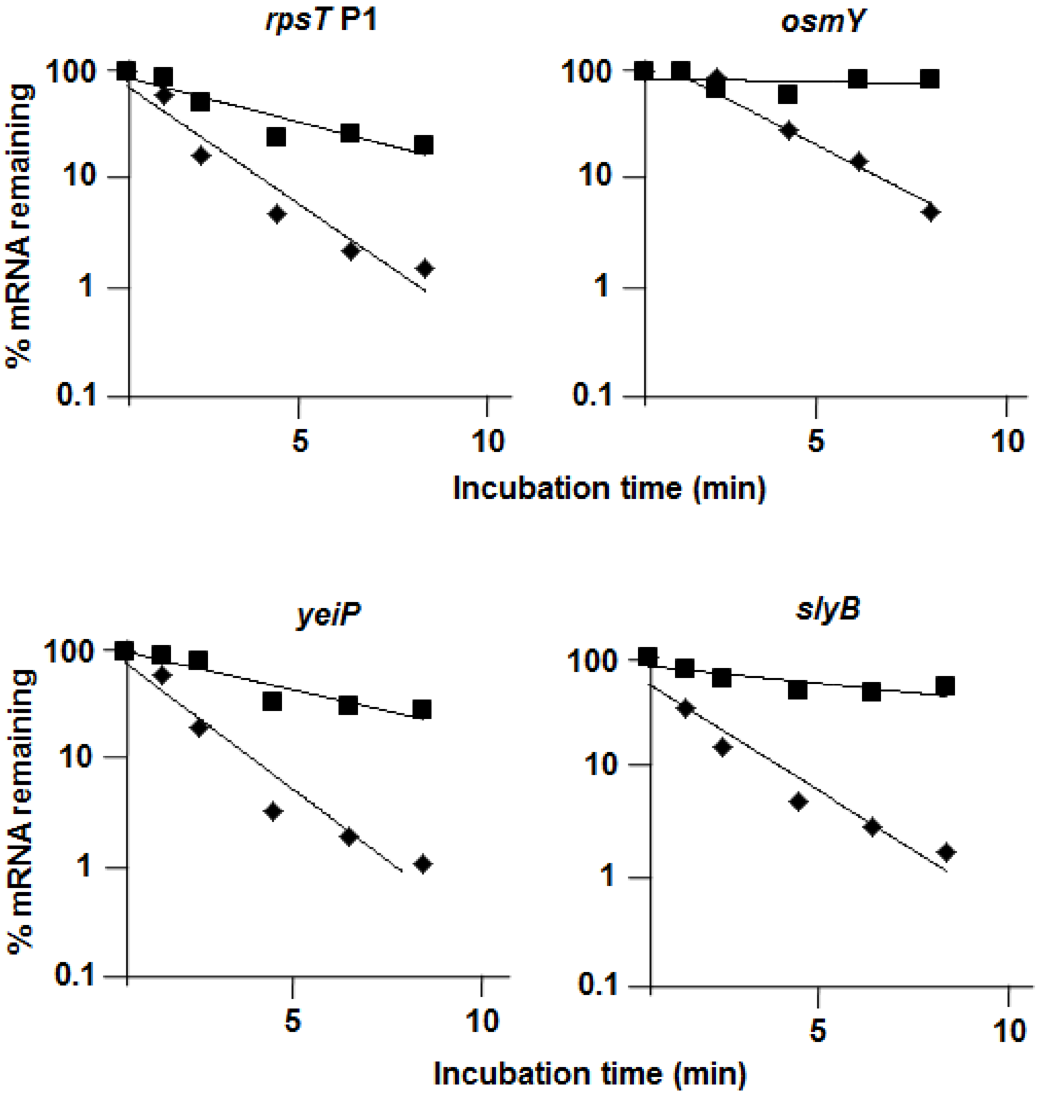

Figure S13. Effect of the *dapF* mutation on the decay rates of RppH target mRNAs

The total RNAs were extracted from the wild-type (diamonds) and the *dapF* mutant strain (squares) at the indicated times after inhibiting transcription by the addition of rifampin. Transcript levels were analyzed by qRT-PCR with primers specific for the *rpsT* P1, *osmY*, *slyB*, *yeiP*, or 16S rRNA. The mRNA levels were normalized to the concentration of 16S rRNA and plotted as a function of time.

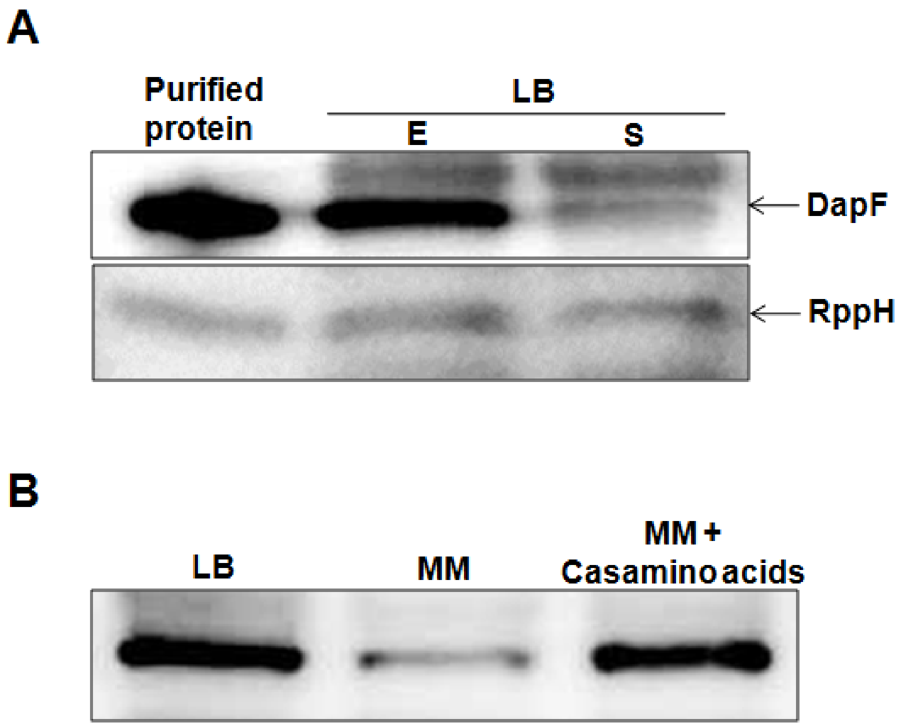

Figure S14. Protein levels of DapF and RppH in *E. coli* cells growing under different conditions

(**A**) Proteins levels of DapF and RppH were determined by Western blot analyses in *E. coli* MG1655 cells at two different growth phases in LB: E, exponential phase; S, stationary phase. Approximately $2x10^8$ cells from each sample were harvested, resuspended in 2x SDS sample buffer and subjected to SDS-PAGE. Western blot analyses were performed using polyclonal antibodies against DapF and RppH raised in rabbit. Purified DapF (20 ng) and RppH (3 ng) were run as loading controls. Representative blots of at least three independent experiments are shown. (**B**) *E. coli* cells were grown in LB, M9 medium (MM), and M9 medium supplemented with 0.5% casamino acids (MM + Casamino acids). When the culture reached an OD_{600} of approximately 1.0, cells from 200 µl of each culture were harvested, and Western blot analysis was performed to determine the DapF expression level.

Supplementary references

1. Blattner, F.R., Plunkett, G., 3rd, Bloch, C.A., Perna, N.T., Burland, V., Riley, M., Collado-Vides, J., Glasner, J.D., Rode, C.K., Mayhew, G.F. *et al.* (1997) The complete genome sequence of *Escherichia coli* K-12. *Science*, **277**, 1453-1462.
2. LaVallie, E.R., DiBlasio, E.A., Kovacic, S., Grant, K.L., Schendel, P.F. and McCoy, J.M. (1993) A thioredoxin gene fusion expression system that circumvents inclusion body formation in the *E. coli* cytoplasm. *Biotechnology (NY)*, **11**, 187-193.
3. Yu, D., Ellis, H.M., Lee, E.C., Jenkins, N.A., Copeland, N.G. and Court, D.L. (2000) An efficient recombination system for chromosome engineering in *Escherichia coli*. *Proc. Natl. Acad. Sci. USA*, **97**, 5978-5983.
4. Reddy, P., Peterkofsky, A. and McKenney, K. (1989) Hyperexpression and purification of *Escherichia coli* adenylate cyclase using a vector designed for expression of lethal gene products. *Nucleic Acids Res*, **17**, 10473-10488.
5. Lee, C.R., Koo, B.M., Cho, S.H., Kim, Y.J., Yoon, M.J., Peterkofsky, A. and Seok, Y.J. (2005) Requirement of the dephospho-form of enzyme IIA^{Ntr} for derepression of *Escherichia coli* K-12 *ilvBN* expression. *Mol. Microbiol.*, **58**, 334-344.
6. Lee, C.R., Cho, S.H., Yoon, M.J., Peterkofsky, A. and Seok, Y.J. (2007) *Escherichia coli* enzyme IIA^{Ntr} regulates the K^+ transporter TrkA. *Proc. Natl. Acad. Sci. USA*, **104**, 4124-4129.
7. Karimova, G., Ullmann, A. and Ladant, D. (2000) *Bordetella pertussis* adenylate cyclase toxin as a tool to analyze molecular interactions in a bacterial two-hybrid system. *Int. J. Med. Microbiol.*, **290**, 441-445.
8. Karimova, G., Ullmann, A. and Ladant, D. (2001) Protein-protein interaction between *Bacillus stearothermophilus* tyrosyl-tRNA synthetase subdomains revealed by a bacterial two-hybrid system. *J. Mol. Microbiol. Biotechnol.*, **3**, 73-82.
9. Karimova, G., Dautin, N. and Ladant, D. (2005) Interaction network among *Escherichia coli* membrane proteins involved in cell division as revealed by bacterial two-hybrid analysis. *J. Bacteriol.*, **187**, 2233-2243.
